# Synaptic retrograde regulation of the PKA-induced SNAP-25 and Synapsin-1 phosphorylation

**DOI:** 10.1186/s11658-023-00431-2

**Published:** 2023-03-03

**Authors:** Aleksandra Polishchuk, Víctor Cilleros-Mañé, Laia Just-Borràs, Marta Balanyà-Segura, Genís Vandellòs Pont, Carolina Silvera Simón, Marta Tomàs, Neus Garcia, Josep Tomàs, Maria A. Lanuza

**Affiliations:** Unitat d’Histologia i Neurobiologia (UHNEUROB), Departament de Ciències Mèdiques Bàsiques, Facultat de Medicina i Ciències de la Salut, Universitat Rovira i Virgili, c/ Sant Llorenç 21, 43201 Reus, Spain

**Keywords:** Neuromuscular junction, Synapse, Neurotransmission, ACh release, SNAP-25, Synapsin-1, PKA subunits, Synaptic activity, Electrical stimulation

## Abstract

**Background:**

Bidirectional communication between presynaptic and postsynaptic components contribute to the homeostasis of the synapse. In the neuromuscular synapse, the arrival of the nerve impulse at the presynaptic terminal triggers the molecular mechanisms associated with ACh release, which can be retrogradely regulated by the resulting muscle contraction. This retrograde regulation, however, has been poorly studied. At the neuromuscular junction (NMJ), protein kinase A (PKA) enhances neurotransmitter release, and the phosphorylation of the molecules of the release machinery including synaptosomal associated protein of 25 kDa (SNAP-25) and Synapsin-1 could be involved.

**Methods:**

Accordingly, to study the effect of synaptic retrograde regulation of the PKA subunits and its activity, we stimulated the rat phrenic nerve (1 Hz, 30 min) resulting or not in contraction (abolished by µ-conotoxin GIIIB). Changes in protein levels and phosphorylation were detected by western blotting and cytosol/membrane translocation by subcellular fractionation. Synapsin-1 was localized in the levator auris longus (LAL) muscle by immunohistochemistry.

**Results:**

Here we show that synaptic PKA Cβ subunit regulated by RIIβ or RIIα subunits controls activity-dependent phosphorylation of SNAP-25 and Synapsin-1, respectively. Muscle contraction retrogradely downregulates presynaptic activity-induced pSynapsin-1 S9 while that enhances pSNAP-25 T138. Both actions could coordinately contribute to decreasing the neurotransmitter release at the NMJ.

**Conclusion:**

This provides a molecular mechanism of the bidirectional communication between nerve terminals and muscle cells to balance the accurate process of ACh release, which could be important to characterize molecules as a therapy for neuromuscular diseases in which neuromuscular crosstalk is impaired.

**Supplementary Information:**

The online version contains supplementary material available at 10.1186/s11658-023-00431-2.

## Background

The neuromuscular system is a complex and interconnected network that links the nervous system with musculature, and their interaction is fundamental for their health and correct functionality. This crosstalk starts at the neuromuscular junction (NMJ) and is mediated by pre- and postsynaptic signals that are regulated by the nerve stimulus and the resulting muscle contraction. This bidirectional regulation is transduced into the synaptic cells and confluent on protein kinases that trigger intracellular pathways, which modulate activity-dependent mechanisms such as neurotransmission and synaptic plasticity. One of these kinases is the protein kinase A (PKA), a cAMP-dependent serine–threonine protein kinase controlling general cellular mechanisms but also synaptic-induced transduction pathways [1, 2] at many synapses including the NMJ [3–7]. However, it is unknown how PKA is regulated by synaptic activity and whether it can be retrogradely regulated by the resulting muscle contraction to promote ACh release and plasticity.

PKA is composed of two catalytic (C) and two regulatory (R) subunits, constituting an inactive tetrameric structure that is activated when cAMP binds to the R subunits and the C subunits are liberated [8–10]. Regarding the structure, there are two classes of PKA holoenzymes, types I and II, which differ in their R subunits (RI and RII; 11, 12). Two catalytic subunits isoforms (Cα, Cβ) and four regulatory subunits isoforms (RIα, RIβ, RIIα, RIIβ) have been reported to be expressed in murine models [13, 14]. R subunits homodimerize through interactions at their N-terminus, generating the holoenzymes RIα2C2, RIβ2C2, RIIα2C2, or RIIβ2C2. Generally, there is no preference for the association between certain R and C subunits [15]. The tissue distribution of these PKA regulatory subunits varies between isoforms; RIα and RIIα are extensively expressed in different tissues, whereas RIβ and RIIβ are found in a more tissue-specific type, concretely RIβ in the nervous system and RIIβ in the liver and hepatic tissue [15–19]. With regard to the NMJ, some regulatory subunits—RIα, RIIα, and RIIβ—have been found at the synaptic area [20–23] and a catalytic subunit, Cβ, has been located in the three cell components of the neuromuscular synapse [20].

Activation of PKA catalytic function depends on the presence of cAMP. This second messenger can bind to the cyclic nucleotide-binding domain (CNB domain) of regulatory subunits, which promotes the release and activation of PKA catalytic subunits. Conversely, in the absence of cAMP, the two regulatory and two catalytic subunits remain together, forming an inactive tetramer [10]. PKA activity is also regulated by its concentration through transcriptional or posttranscriptional changes in the synthesis or degradation of its subunits [20, 24–26]. Furthermore, the intracellular targeting and compartmentalization of the subunits of PKA are controlled through association with A-kinase-anchoring proteins (AKAPs), which have a motif in their structure with high affinity to PKA regulatory subunits, so they remain stacked together [10]. AKAPs control the optimal placing of PKA close to the temporally regulated pools of cAMP, and moreover, AKAPs target PKA to their specific substrates and assemble multiprotein signal complexes [27–29]. The role of AKAPs in the specificity of cAMP signal transduction seems to be related to the regulation of the PKA subunits dynamics between membrane and cytosol [15, 28], in particular AKAP150 at the NMJ [20].

With the aim to investigate the influence of the pre- and postsynaptic activity on the neurotransmission modulation at the presynaptic component of the NMJ, we have chosen two PKA targets, synaptosomal associated protein of 25 kDa (SNAP-25) and Synapsin-1, in accordance with their implication with acetylcholine release and their presynaptic location [30, 31]. SNAP-25 is a key protein for the fusion of synaptic vesicles with the plasma membrane during exocytosis [32]. PKA phosphorylates SNAP-25 on T138 (pSNAP-25 T138) [33, 34], and this phosphorylation occurs in response to synaptic activity and is involved in controlling the size of the releasable vesicle pools [30]. PKA also phosphorylates Synapsin-1 at S9, which is a cytoplasmic surface synaptic vesicle protein [35]. Its phosphorylation on S9 decreases actin binding, allowing synaptic vesicles to mobilize to the releasable pool [31]. Although SNAP-25 and Synapsin-1 are phosphorylated by PKA, and this phosphorylation is promoted by synaptic activity, there is no information about the synaptic activity-dependent PKA regulation, and this regulation is unknown at the NMJ. This is an important issue as, at the NMJ, PKA signaling enhances neurotransmitter release [36, 37]. Furthermore, it is unexplored whether synaptic activity-induced muscle contraction regulates PKA and its downstream pathway. This is also an important question to better understand the bidirectional communication occurring at the NMJ that contributes to maintain the synapse functional and healthy. We know that synaptic activity-induced muscle contraction regulates protein kinase C (PKC) functions on several substrates from the synaptic exocytotic machinery through the BDNF/TrkB receptor pathway [38–40]. Furthermore, PKC and PKA collaborate in the regulation of the ACh release at the NMJ [37, 41], which strongly suggests a retrograde regulation on PKA.

Therefore, here we hypothesize that presynaptic stimulation and the induced muscle contraction differently modulate PKA catalytic and regulatory subunits to balance synaptic function and regulate kinase functions on several substrates from the synaptic exocytotic machinery. This study demonstrates that presynaptic activity and the resulting muscle contraction coordinately regulate the PKA signaling pathway at the NMJ and their phosphorylating activity on its relevant synaptic substrates pSynapsin-1 and pSNAP-25 T138. These findings add to our knowledge of the bidirectional communication between nerve terminals and muscle fibers to enhance acetylcholine release through PKA-phosphorylation targets at the NMJ.

## Methods

### Animal care

Male and female adult Sprague Dawley rats (30–40 days; Criffa, Barcelona, Spain; RRID: RGD_5508397) were cared for in accordance with the European Community Council Directive guidelines for the humane treatment of laboratory animals. For samples of tissue harvest, the animals were euthanized with a lethal dose of 4% tribromoethanol (Sigma-Aldrich). For each type of experimental condition, at least three animals (*n* ≥ 3) were used as a biological iteration. All animal work was approved by the Ethics Committee of Animal Experimentation of the Universitat Rovira i Virgili (reference number 10760).

### Antibodies

For western blotting, primary and secondary antibodies were obtained from the following sources and were used with the following concentrations (Table 1). Before applying western blotting technique, we analyzed the specificity of the primary antibodies. All of them showed the specific bands with expected molecular weights (Figs. 2A, 4A, 5A).

**Table 1:** Antibodies

Target	Epitope	Source	Company (cat. no.)	Dilution
Cα	Hu Cα C-terminus	Ms mAb	Santa Cruz (sc-28315)	1/1000
Cβ	Hu Cβ C-terminus	Rb pAb	Santa Cruz (sc-904)	1/1000
RIα	Hu RIα residues 1–381	Ms mAb	Santa Cruz (sc-136231)	1/1000
RIβ	Hu RIβ C-terminus	Ms mAb	Santa Cruz (sc-100414)	1/1000
RIIα	Ms RIIα C-terminus	Rb pAb	Santa Cruz (sc-909)	1/1000
RIIβ	Hu RIIβ residues 21–110	Ms mAb	Santa Cruz (sc-376778)	1/800
SNAP-25	Hu SNAP-25 residues around Gln116	Rb mAb	CST (5309)	1/1000
pSNAP-25 (T138)	Hu SNAP-25 residues around T138	Rb pAb	Biorbyt (orb163730)	1/1000
Synapsin-1	Hu Synapsin-1a,b	Rb pAb	AB1543 Chemicon	1/1000 1/500
pSynapsin-1 (S9)	Hu Synapsin-1 residues around S9	Rb pAb	CST (2311S)	1/1000
AKAP150	Rat AKAP150 residues 428–449	Rb pAb	Millipore (07-210)	1/1000
GAPDH	Rb GAPDH	Ms mAb	Santa Cruz (sc-32233)	1/4000
ATPase	Chicken ATPase residues 27–55	Ms mAb	DSHB (a6f)	1/2000
Syntaxin	Rat Syntaxin	Ms mAb	Millipore (S0664)	1/1000
Secondary antibodies	Anti-Rb conjugated HRP	Dk pAb	711-035-152	1/10000
	Anti-Ms conjugated HRP	Rb pAb	A9044	1/10000
	Anti-Ms conjugated TRITC	Dk pAb	715-025-151	1/1000
	Anti-Rb conjugated Alexa fluor 488	Dk pAb	A21206	1/1000
	α-Bungarotoxin conjugated Alexa Fluor 647		B35450	1/1000
	α-Bungarotoxin conjugated TRITC		T1175	1/1000

Antibodies used in this study and procedure specifications

*Dk* donkey, *Hu* human, *mAb* monoclonal antibody, *Ms* mouse, *pAb* polyclonal antibody, *Rb* rabbit

### Reagents

To block muscle stimulation μ-conotoxin GIIIB (#C-270, Alomone Labs Ltd, Jerusalem, Israel) was used. This toxin inhibits sarcolemmal voltage-dependent sodium channels (VSDCs) without affecting synaptic ACh release or ACh signaling [42]. It was supplied as lyophilized powder of > 99% purity. μ-Conotoxin GIIIB was 150 μM stock, and working concentration was 1.5 μM in Ringer’s solution [mM: NaCl 137, KCl 5, CaCl_2_ 2, MgSO_4_ 1, NaH_2_PO_4_ 1, NaHCO_3_ 12, glucose 12.1, and DMSO 0.1%, oxygenated with O_2_:CO_2_ (95:5)].

PKA activity was blocked with *N*-[2-((*p*-Bromocinnamyl)amino)ethyl]-5-isoquinolinesulfonamide dihydrochloride (H-89, Calbiochem). H-89 was made as 10 mM stock and used at 10 μM diluted in Ringer’s solution with DMSO.

All chemicals were diluted in Ringer’s solution, and both control and drug-containing solutions contained 0.1% dimethyl sulfoxide (DMSO) as the vehicle.

### Tissue dissection and treatment

As a typical model to study the development and function of the NMJ [43–45], diaphragm muscle was dissected with special care to preserve phrenic nerve connectivity. Isolated nerve–muscle preparations were immersed in Ringer’s solution and maintained at 26 °C.

**Fig. 1:**
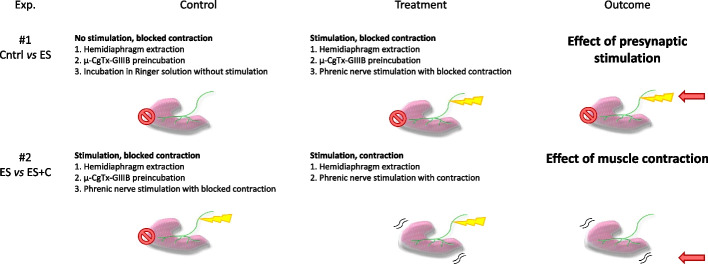
Design of experimental treatment for the study of effects of presynaptic activity and nerve-induced muscle contraction. μ-CgTx-GIIIB, μ-conotoxin GIIIB

One hemidiaphragm was used as a treatment, and the other served as its paired untreated control. All treatments were performed ex vivo. Muscles were stimulated through the phrenic nerve at 1 Hz, which allows the maintenance of different tonic functions without depleting synaptic vesicles, for 30 min using the A-M Systems 2100 isolated pulse generator (A-M System) as in previous studies [38–40]. We designed a protocol of stimulation that preserves the nerve stimulation and the associated neurotransmission mechanism. This method prevents other mechanisms associated with non-nerve-induced (direct) muscle contraction [46–48]. To verify muscle contraction, a visual checking was done. Two main experiments were performed to distinguish the effects of synaptic activity from those of muscle activity (Fig. 1).

1. Presynaptic stimulation (Ctrl versus ES): to show the impact of the synaptic activity, we compared presynaptically stimulated muscles whose contraction was blocked by μ-CgTx-GIIIB with nonstimulated muscles also incubated with μ-CgTx-GIIIB to control for nonspecific effects of the blocker.
2. Contraction (ES versus ES + C): to estimate the effect of nerve-induced muscle contraction, we compared stimulated/contracting muscles with stimulated/noncontracting muscles whose contraction was blocked by μ-CgTx-GIII. By comparing the presynaptic stimulation with or without postsynaptic activity, we separate the effect of contraction. However, one should consider that postsynaptic contraction experiments also contain presynaptic activity.

In the experiments that needed only stimulation without contraction, μ-CgTx-GIIIB was used (see “Reagents”). Nevertheless, before immersing these muscles in μ-CgTx-GIIIB, a visual checking of the correct contraction of the muscle was done [39].

Furthermore, to assess the effect of PKA blocking, three different experiments have been performed:

1. To estimate the effect of PKA inhibition under synaptic activity, we compared presynaptically stimulated muscles whose contraction was blocked by μ-CgTx-GIIIB with and without H-89: ES versus ES + H-89.
2. To show the impact of the PKA inhibition under muscle contraction, we compared stimulating and contracting muscles with and without H-89: (ES + C) versus (ES + C) + H-89.
3. To demonstrate if degradation or redistribution along the axon is involved, the diaphragm muscle was dissected with special care to preserve phrenic nerve connectivity. We compared stimulating and contracting muscles with and without protease inhibitor (Prot.Inh.) cocktail 1% (10 μl/ml; Sigma, Saint Louis, MO, USA): (ES + C) versus (ES + C) + Prot.Inh.

### Sample processing by western blotting and fractionation

Whole-cell lysate samples were immediately frozen after treatment completion. Membrane/cytosol fractionated lysates were processed immediately after the treatment without a freezing step. Further details of homogenization and western blotting technique can be consulted in Cilleros-Mañé et al. [41].

The densitometry of the bands was obtained with ImageJ software. The integrated optical density of the bands was normalized with respect to: (1) the background values; and to (2) the total protein transferred on PVDF membranes, measured by total protein analysis (Sypro Ruby protein blot stain, Bio-Rad [49]). The relative variations between the experimental samples and the control samples were calculated from the same membrane image. All presented data derive from densitometry measurements made of three to ten separate replicates, plotted against controls. Data quantification was performed blindly.

### Proteomics

Diaphragm preparations were collected and processed to obtain proteome data. Protein identification, filtering, and quantification were performed by a specialized Centre for Omic Sciences at the Universitat Rovira i Virgili. In brief, protein identification and quantification was performed on Proteome Discoverer software v.1.4.0.288 (Thermo Fisher Scientific, CA, USA) by Multidimensional Protein Identification Technology (MudPIT) combining the eight raw data files obtained from each fraction of the sample. For protein identification, all MS and MS/MS spectra were analyzed using Mascot search engine (v.2.5). The workflow was set up using two different Mascot node combing *Rattus norvegicus* databases (29,955 entries) and contaminants database (247 entries), both searches assuming trypsin digestion. The false discovery rate (FDR) and protein probabilities were calculated by Percolator. For protein quantification, the ratios between each TMT label against 126-TMT label were used and quantification results were normalized on the basis of protein median. The results are a ratio of reporter ion abundance, and they are dimensionless.

### Immunohistochemistry

Synapsin-1 was localized in the levator auris longus (LAL) muscle by immunohistochemistry (IHC) in accordance with the protocol presented in Cilleros-Mañé et al. [41]. LAL muscles from the same animals that we used for western blot were used to perform this technique (*n* = 3).

As a control, primary antibodies were omitted from some muscles during the immunohistochemical procedures. These control muscles never exhibited positive staining. In double-staining protocols, omitting either one of the two primary antibodies completely abolished the corresponding staining and there was no cross-reaction with the other primary antibody. At least three muscles were used as negative controls.

A laser-scanning confocal microscope (Nikon TE2000-E) was used to study immunolabeled NMJs from the whole-mount muscles [41]. FIJI (ImageJ) software was used to perform 3D colocalization analyses from confocal stacks. The Pearson correlation coefficient (*r*) was used for quantitative analysis of colocalization. This statistic coefficient provides the overall association of two probes in an image. Images were assembled using Adobe Photoshop software (Adobe Systems, San Jose, CA), and neither the contrast nor brightness was modified.

### Statistical analysis

At least three animals (*n* ≥ 3) were used as biological replicates for every experiment detailed previously. Thus, all experiments were carried out at least in three biological replicates, and each one of these was assessed in three technical replicates. Sample size to optimize the number of used animals was calculated using the previously established criteria [50, 51]. The results are presented as ratios or percentages of treatment to control (mean ± SEM). Shapiro–Wilk test was used to test sample normality. Statistical difference was determined with paired Student’s *t*-test or its nonparametric alternative Wilcoxon test (GraphPad Prism, San Diego, USA). Each dot in the bars of the graphs represents the mean result of one animal. The significance threshold was *p < 0.05, **p < 0.01, and ***p < 0.001.

## Results

### Pre- and postsynaptic activity modulation of catalytic and regulatory PKA subunit protein levels

**Fig. 2:**
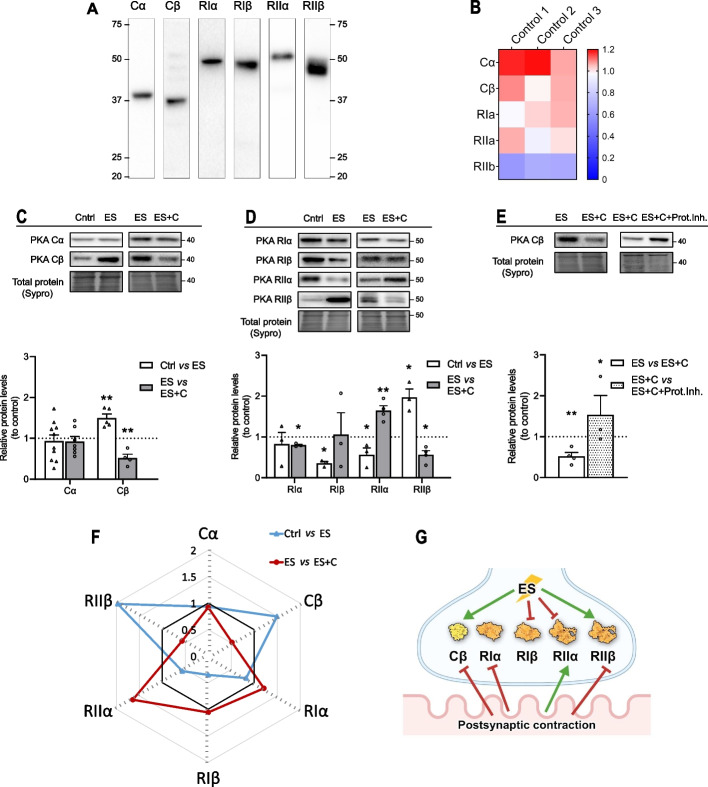
Pre- and postsynaptic activities modulation of PKA catalytic and regulatory subunits protein levels. **A** Western blot analysis and data quantification of diaphragm samples showing specificity of the anti-Cα, -Cβ, -RIα, -RIβ, -RIIα, and -RIIβ antibodies to show protein bands appear in the correct molecular weight. **B** Heatmap from proteomic data of relative abundance of PKA subunits in the rat diaphragm samples in basal conditions. **C**, **D** Western blot analysis of protein levels after treatment with presynaptic stimulation without contraction—Ctrl versus ES—and with muscle contraction—ES versus ES + C. **C** Catalytic PKA Cα and Cβ subunits. **D** Protein kinase A regulatory subunit Iα/Iβ/IIα/IIβ. Data are expressed relative to control (mean ± SD). *p < 0.05, **p < 0.01, and ***p < 0.001 versus the corresponding control. **E** Pre- and postsynaptic activities modulation of PKA catalytic subunit protein levels (Cβ). Data are expressed relative to control (mean ± SD). *p < 0.05, **p < 0.01, and ***p < 0.001 versus the corresponding control. Prot.Inh., protease inhibitor cocktail. **F** Effect of presynaptic activity and postsynaptic muscle contraction on the PKA subunits. The diagram is a graphic representation that collectively shows the patterns of changing of the PKA subunits concentrations during presynaptic activity (Ctrl versus ES—blue), contraction abolished by µ-conotoxin GIIIB, and contraction (ES versus ES + C—red). Data are expressed relative to control (black line) (mean). **G** Graphical representation of the results. Cα/β, protein kinase A catalytic subunit α/β; RIα/RIβ/RIIα/RIIβ, protein kinase A regulatory subunits Iα/Iβ/IIα/IIβ

Firstly, we analyzed whether neuromuscular activity regulates PKA subunit protein levels. We developed an ex vivo experimental system to distinguish the effect of synaptic activity from muscle contraction (Fig. 2; see Additional file 1 for original blots). When we refer to synaptic activity, we include the presynaptic stimulus, synaptic transmission, and the generation of a restricted endplate potential due to ACh signaling. These effects were determined by comparing nonstimulated muscles (Ctrl, control) with stimulated muscles with their contraction blocked with µ-CgTx-GIIIB (electrically stimulated, ES). On the other hand, when we refer to the nerve-induced muscle contraction, we include membrane depolarization of the muscle fiber involving voltage-dependent sodium channels and the resulting myofiber contraction. These effects were determined by comparing stimulated muscles without contraction (Ctrl versus ES, electrically stimulated) with stimulated muscles with contraction (ES versus ES + C, stimulation and contraction).

To study the protein levels of the PKA catalytic (C) and regulatory (R) subunits, we selected the antibodies that showed high specificity for the corresponding protein at the predicted molecular weight (in kDa): Cα 40, Cβ 40, RIα 48, RIβ 51, RIIα 50, and RIIβ 53 in the rat diaphragm (Fig. 2A) [52, 53]. Antibody specificity was previously validated through knockout cell lysates and with cell lines that do not express the target PKA subunit [20]. To estimate the distribution of the PKA subunits in the rat’s diaphragm, we have used the proteomic technique. Mouse models exhibit four isoforms of R subunits (RIα, RIβ, RIIα, RIIβ), and two isoforms of C subunits (Cα, Cβ). The Cγ gene is exclusive to primates [13, 14]. In accordance with these data, we detected with proteomics relatively high levels of both Cα and Cβ subunits in the rat diaphragm samples (Fig. 2B). The expression of the R subunits varies across tissues, and they have unique functions in cell growth and differentiation [16, 17]. RIα and RIIα are widely expressed, while RIβ is primarily found in the nervous system and RIIβ in adipose and liver tissues [15–18]. Indeed, RIβ was not detectable with proteomics due to low level of expression in the diaphragm, and the concentration of RIIβ was very low in the samples (Fig. 2B). However, using western blotting technique we were able to identify each subunit of PKA.

With the anti-PKA antibodies validated, we studied whether the presynaptic activity and the resulting muscle contraction regulate the protein levels of C and R subunits. The PKA catalytic subunits Cα and Cβ reacted in different ways to synaptic activity and muscle contraction (Fig. 2C). PKA Cα levels were unresponsive to any nerve stimulation, whereas Cβ levels increased by the presynaptic stimulus (Ctrl versus ES) and decreased by muscle contraction (ES versus ES + C). The molecular mechanism of Cβ protein changes during NMJ activity is unknown. To demonstrate if the contraction-induced decrease in Cβ levels is a result of degradation or redistribution along the axon, we compared stimulating and contracting muscles with and without a protease inhibitor (Prot.Inh.) cocktail 1% (10 μl/ml; Sigma, Saint Louis, MO, USA). By analyzing the data, we found an increase in Cβ levels during nerve-induced muscle contraction treated with protease inhibitor cocktail (Fig. 2E). Thus, the decrease shown during postsynaptic muscle contraction was reversed. According to this finding, we can conclude that the decrease of Cβ levels during muscle contraction was induced following proteasomal activity and activity-induced degradation of the subunit. In addition, the opposed actions of pre- and postsynaptic activities on Cβ would explain why this subunit is unaltered when both activities are present, i.e., in response to presynaptic stimulus with contraction. The difference between subunits argues in favor of different mechanisms controlling each catalytic subunit.

The PKA regulatory subunits also responded differently to presynaptic stimulus (Fig. 2D). RIα remained stable, RIβ and RIIα were decreased, and RIIβ was increased. Also, regulatory subunits do not follow a unique trend after the nerve-induced muscle contraction. RIα decreases, RIβ remains stable, RIIα increases, and RIIβ is decreased as a consequence of the postsynaptic muscle response. These results show that each regulatory subunit has its own pathway to regulate its levels. It can be summarized that the presynaptic stimulation and nerve-induced muscle contraction contrarily regulate Cβ, RIIα, and RIIβ. This suggests that the two regulatory subunits RII (α and β) differentially regulate Cβ: RIIα would contribute to increasing or decreasing PKA activity in presynaptic (Ctrl versus ES) and postsynaptic (ES versus ES + C) activities, respectively, and RIIβ would contribute to decreasing or increasing PKA activity in presynaptic or postsynaptic activities, respectively.

In summary, all C and R subunits can have their own response to neuromuscular synapse activity events, and a modulatory influence of the postsynaptic activity on the PKA activity can be inferred. Figure 2E shows a spider graph of the protein level changes of the PKA subunits to facilitate interpretation of these multiple changes, and Fig. 2F shows a graphical summary of the findings.

### Regulation of cytosol–membrane PKA subunits translocation by pre- and postsynaptic activities

**Fig. 3:**
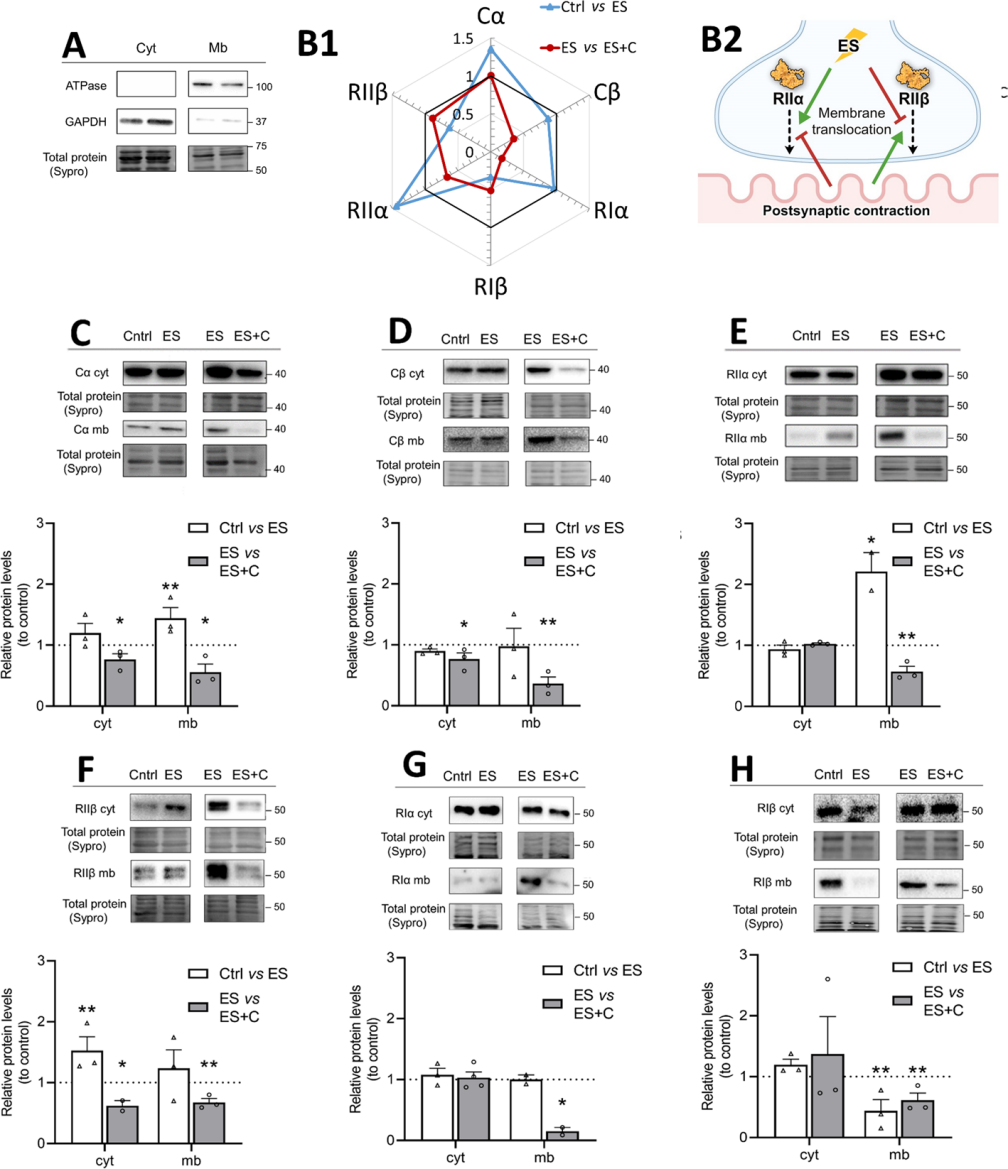
Regulation of cytosol–membrane PKA subunits translocation by pre- and postsynaptic activities. **A** The purity of the subcellular fractionation was validated by western blotting of the fraction-specific proteins GAPDH for cytosol and Na+/K+-ATPase for membrane. **B1** Effect of presynaptic stimulus and postsynaptic muscle contraction on the ratio of membrane (mb)/cytosol (cyt) fractions of the PKA subunits. The diagrams are graphic representations that collectively show the patterns of changing of the ratio mb/cyt fractions of the PKA subunits during presynaptic activity (Ctrl versus ES—blue), contraction abolished by µ-conotoxin GIIIB, and contraction (ES versus ES + C—red). Data are expressed relative to control (black line) (mean). **B2** Graphical representation of the results. **C**–**H** Western blot analysis and data quantification of catalytic (Cα, Cβ) and regulatory (RIα, RIβ, RIIα, RIIβ) PKA subunits in the cytosol and membrane fractions of the diaphragm muscle. Protein levels are studied after treatment with presynaptic stimulation without contraction—Ctrl versus ES and with muscle contraction—ES versus ES + C. Data are expressed relative to control (mean ± SD). *p < 0.05, **p < 0.01, and ***p < 0.001 versus the corresponding control. Cα/β, protein kinase A catalytic subunit α/β; GAPDH, glyceraldehyde-3-phosphate dehydrogenase; RIα/RIβ/RIIα/RIIβ, protein kinase A regulatory subunits Iα/Iβ/IIα/IIβ; cyt, cytosol; mb, membrane

Because the subcellular distribution of the PKA subunits contributes to the regulation of PKA action, we next examined how pre- and postsynaptic activities redistribute the PKA subunits between the membrane and cytosol fraction. Figure 3 shows the relative level of protein of each PKA subunit in the cytosol and membrane fraction after the specific stimulation treatment in the diaphragm muscle. Bars represent the relative protein levels (i.e., each particular control defined as 1 and treatment calculated in relation to control). Additionally, all data were normalized to the total protein loaded. We used Na+/K+-ATPase and GAPDH as markers to confirm the purity of the subcellular fractionation. Na+/K+-ATPase and GAPDH were highly enriched in their fractions, of membrane and cytosol, respectively (Fig. 3A). To facilitate analysis of the data, Fig. 3B1 shows a spider graph of the observed membrane/cytosol ratios and Fig. 3B2 a graphical abstract for interpretation. As previously found in skeletal muscle [20], the results show that both C and R subunits were predominantly located in the cytosol fraction.

The Cα (Fig. 3C) and Cβ (Fig. 3D) did not change their levels in cytosolic fraction during presynaptic stimulus (Ctrl versus ES) indicating that it did not induce their translocation. It is interesting to note that Cα increased in the membrane during the presynaptic stimulus, a modulation that was not detected in whole-cell samples probably due to the majority of Cα being associated with the cytosol. However, both C subunits decreased their level in both cytosol and membrane fractions after the nerve-induced muscle contraction (ES versus ES + C), indicating that the contraction-induced regulation results in a general downregulation in all the compartmentalized fractions without affecting translocation between compartments.

When regulatory subunits are analyzed after presynaptic stimulation, we found that presynaptic stimulus (Ctrl vs ES) did not change the cytosolic levels of any of the R subunits except for RIIβ, which increased, which would contribute to maintaining the holoenzyme in the cytosol and negatively regulating the catalytical action of the PKA (Fig. 3E–H). In addition, in this condition of stimulation, we observed an increase in RIIα in the membrane that would contribute to enhancing the catalytical action of PKA. On the contrary, RIβ decreased in the membrane, which would contribute to downregulating the catalytical action of PKA.

Analyzing regulatory II subunits in the contraction condition (ES versus ES + C), we found that RIIα and RIIβ (Fig. 3E, F) were differently affected. RIIα decreased in the membrane, which would oppose the catalytic action of PKA. This regulatory mechanism, however, might not be so strong as it is not reflected in an increase in the cytosolic fraction. In contrast, RIIβ decreased in the cytosol, which would contribute to the release of the catalytic subunits from the holoenzyme and therefore increase their activity. In addition, RIIβ also decreased in the membrane fraction according to the global reduction of the subunit in this condition.

Finally, we found that regulatory I subunits are regulated only during nerve-induced muscle contraction, although RIβ also decreased in the membrane as a result of the presynaptic stimulation, which would oppose the catalytic action of PKA (Fig. 3G, H). RIα did not change its cytosolic levels during nerve-induced muscle contraction (ES versus ES + C) but decreased in the membrane (Fig. 3G), which would oppose the catalytic action of PKA. This regulatory mechanism, however, might not be so strong as it is not reflected in an increase in the cytosolic fraction.

In summary: (i) electrical stimulation results in a moderate increase of total Cβ and a redistribution of both RIIα (less expression and displacement to the membrane) and RIIβ (increased total expression mainly in the cytosol); and (ii) after the nerve-induced muscle contraction, there is a total reduction of the Cβ both in membrane and cytosol as well as an inversion of the RIIα and RIIβ protein level and subcellular distribution that suggests a balance between both regulatory isoforms to control PKA activity.

### Postsynaptic contraction modulates AKAP150

**Fig. 4:**
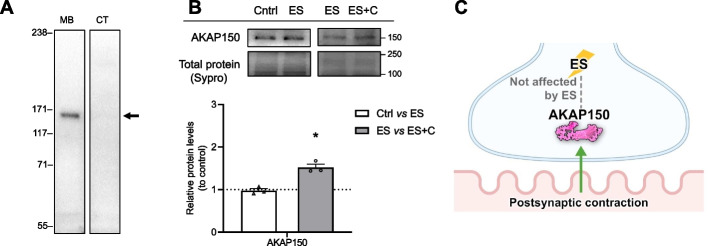
Pre- and postsynaptic activities modulate AKAP150 protein levels. **A** Western blot analysis and data quantification of diaphragm samples showing specificity of the anti-AKAP150 antibodies to show protein bands appear in the correct molecular weight. **B** Western blot analysis and data quantification of AKAP150 protein levels in the diaphragm muscle after pre- and postsynaptic activities. **C** Graphical representation of the results. Data are expressed relative to control (mean ± SD). *p < 0.05, **p < 0.01, and ***p < 0.001 versus the corresponding control. AKAP150, A kinase anchor protein 150; cyt, cytosol; mb, membrane

After determining that neuromuscular activity regulates the association of PKA with the cytosol and membrane compartments, we studied whether the anchor protein AKAP150 could be involved because it participates in the neuromuscular muscarinic-induced PKA signaling [20]. We used an anti-AKAP150 antibody, which reacted with a unique band of the predicted 150 kDa molecular weight (Fig. 4A). This antibody was raised against the peptide sequence corresponding to the amino acids 428–449 of rat AKAP150. Blasting this sequence against a rat database showed 100% identity with AKAP150 (Uniprot sequence P24587), whereas the other hits presented gaps and less than 60% identity and their molecular weight did not correspond with the observed band (40–86 kDa versus the observed 150 kDa). In the total fraction, AKAP150 protein levels were not affected by presynaptic stimulus (Ctrl versus ES) (Fig. 4B). Nevertheless, muscle contraction (ES versus ES + C) increased the levels of this scaffold protein, indicating a possible retrograde regulation. Figure 4C shows a graphical summary of the findings.

### Pre- and postsynaptic activities module SNAP-25 and Synapsin-1 phosphorylation through PKA

**Fig. 5:**
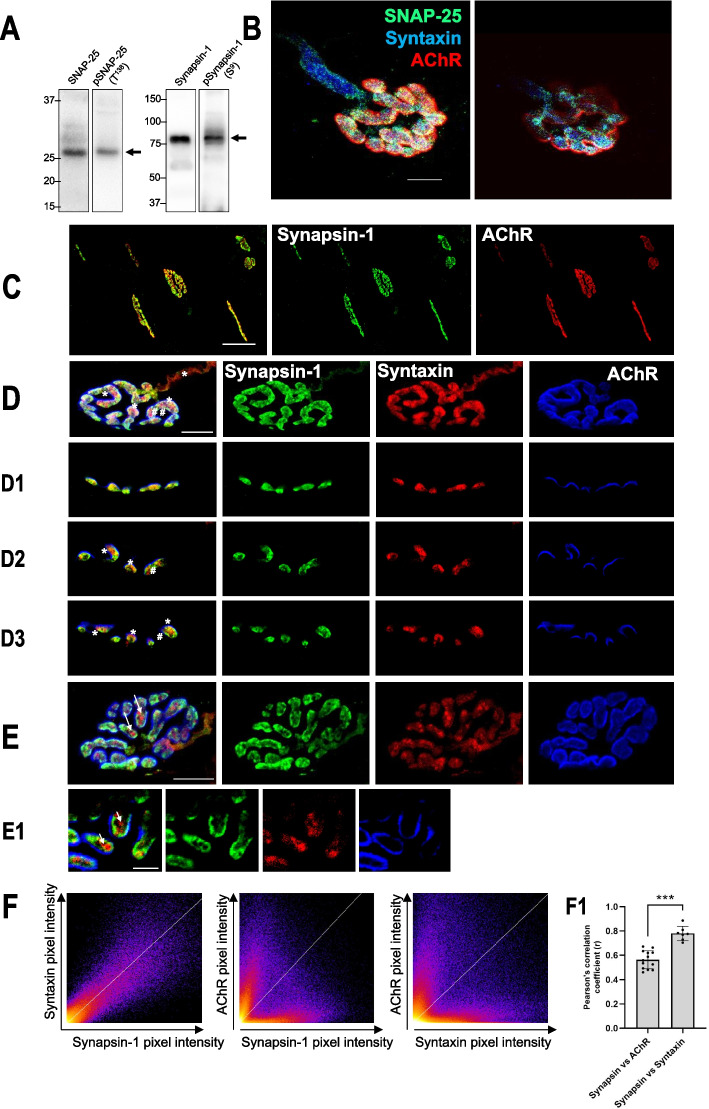
Synapsin-1 localization at the adult NMJ. **A** Western blot analysis and data quantification of diaphragm samples showing specificity of the anti-SNAP25/pSNAP25 and anti-Synapsin-1/pSynapsin-1 antibodies to show protein bands appear in the correct molecular weight. **B**–**E1** Neuromuscular junctions of levator auris longus (LAL) muscle visualized at confocal microscopy. **B** Confocal images of a NMJ with triple labeling: SNAP-25 (green), Syntaxin (blue), and AChR (red). Scale bar, 10 μm. **C** Confocal images of multiple NMJ stained with Synapsin-1 (green) and AChR (red). Scale bars, 50 μm. **D** Confocal images of an NMJ with triple labeling: Synapsin-1 (green), Syntaxin (red), and AChR (blue). Scale bar, 10 μm. **D1**–**D3** Confocal optical section of the NMJ shown in **B**. Scale bars, 10 μm. “#” shows areas of the nerve terminal rich in Synapsin-1 and poor in Syntaxin. “*” shows areas of the nerve terminal rich in Syntaxin and poor in Synapsin-1. **E** Confocal images of a NMJ with triple labeling: Synapsin-1 (green), Syntaxin (red), and AChR (blue). Scale bar, 10 μm. **E1** Scale bar, 3 μm. Arrows show areas of the nerve terminal rich in Syntaxin and poor in Synapsin-1. **F** Quantitative colocalization represented as heatmap of the intensity of between anti-Synapsin-1 labeling, presynaptic anti-Syntaxin labeling and postsynaptic AChR labeling. **F1** Average Pearson’s correlation coefficient of the colocalization between Synapsin-1 versus Syntaxin or Synapsin-1 versus AChRs. ***p < 0.001 versus the corresponding comparison

To further explore the neuromuscular activity regulation on the PKA signaling and focus on the presynaptic effects, we evaluated the phosphorylation of the PKA substrates SNAP-25 and Synapsin-1 (Fig. 5). Both SNAP-25 and Synapsin-1 are relevant presynaptic PKA targets involved in ACh release. SNAP-25 is a SNARE component that is crucial for neurotransmission, and it is phosphorylated by PKA on threonine 138 (T138), reducing SNARE complex stability and promoting synaptic vesicles recycling and refilling. Synapsin-1 is a key molecule involved in the link between synaptic vesicles and the actin cytoskeleton. Phosphorylation of Synapsin-1 by PKA on serine 9 (S9) causes the dissociation of the protein from the vesicle membrane, allowing diffusion of the vesicles to the periphery of the nerve terminals and enhancing their rate of recycling.

The antibodies against SNAP-25 and Synapsin-1 and their phosphorylated forms detected a band of 28 kDa and 77 kDa, respectively (Fig. 5A). SNAP-25 has been described as a presynaptic molecule at the NMJ [39, 41]. Here we confirm this location (Fig. 5B). With the aim of studying the location of Synapsin-1 in the NMJ, we used fluorescent immunohistochemistry and confocal microscopy. Synapsin-1 is located in the synaptic area of the skeletal muscle. Figure 5C shows that Synapsin-1 (in green) was located within all the endplates observed (labeled with α-bungarotoxin in red). Second, to discover more specifically where Synapsin-1 is positioned we used well-known markers of the pre- and postsynaptic compartment of the NMJ. Labeled Syntaxin and α-bungarotoxin were used to localize the presynaptic and postsynaptic sites, respectively. Figure 5D shows an NMJ stained with triple labeling: Synapsin-1 in green, Syntaxin in red, AChR in blue. As expected, Synapsin-1 was clearly found in the presynaptic component, colocalized with Syntaxin-1 over AChRs location. The lack of overlap between AChR and Synapsin-1 can be clearly seen in the confocal optical sections (D1–D3). Molecular quantitative colocalization was performed selecting NMJ endplates as the region of interest and excluding axons (Fig. 5F). The average Pearson’s correlation of Synapsin-1 versus Syntaxin showed a strong association (*r* = 0.78 ± 0.06), and that of Synapsin-1 versus nAChRs showed a poor association (*r* = 0.56 ± 0.11) (Fig. 5F1). Thus, Synapsin-1 and Syntaxin colocalize and are evidently in the presynaptic compartment over the postsynaptic gutters. Finally, images show that the presynaptic label of Synapsin-1 and Syntaxin are not completely coincident (Fig. 5E). Synapsin-1 was less present in the terminal axon than Syntaxin (asterisk in Fig. 5D). Interestingly, the distribution of Synapsin-1 and Syntaxin was also different in the nerve terminal. “#” shows areas of the nerve terminal rich in Synapsin-1 and poor in Syntaxin (Fig. 5C, D; arrows in E). Asterisk shows areas of the nerve terminal rich in Syntaxin and poor in Synapsin-1 (Fig. 5D1–D3; arrows in E–E1). In conclusion, Synapsin-1 has a location in the nerve terminal that differs from Syntaxin’s location, which probably is representative of its function. The location of SNAP-25 and Synapsin-1 in the nerve terminal of the NMJ facilitates the interpretation of the PKA subunit results to explain the regulation of the PKA activity on the neurotransmitter release at the NMJ. In accordance with the data from IHC technique (Fig. 5), Synapsin-1 and SNAP-25 being located in the presynaptic terminal of the NMJ reinforces that the following biochemical results are presynaptic. However, one should consider that PKA might also be active in the other cell components of the NMJ and that muscle or glial PKA activity may induce some signaling and influence the presynaptic nerve site [20].

**Fig. 6:**
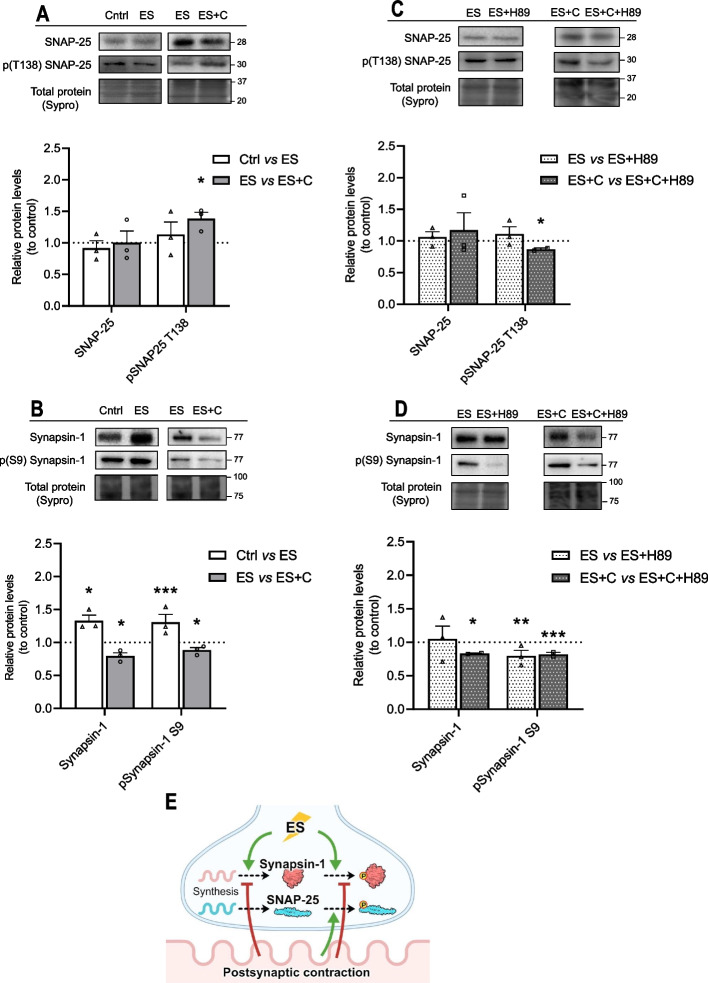
Pre- and postsynaptic activities modulation of the PKA targets synthesis and phosphorylation. Western blot analysis and data quantification of the phosphorylated and total levels of **A** SNAP-25 and **B** Synapsin-1 in the diaphragm muscle after presynaptic stimulus (Ctrl versus ES) and nerve-induced muscle contraction (ES versus ES + C). Western blot analysis and data quantification of the protein levels and phosphorylation of **C** SNAP-25 and **D** Synapsin-1 in the diaphragm muscle after (ES) and (ES + C) with PKA inhibition with H-89. **E** Graphical representation of the results. Data are expressed relative to control (mean ± SD). *p < 0.05, **p < 0.01, and ***p < 0.001 versus the corresponding control. pSNAP-25, Thr138-phosphorylated synaptosomal-associated protein 25; SNAP-25, synaptosomal-associated protein 25; pSynapsin-1, Ser9-phosphorylated Synapsin-1

Figure 6A shows that SNAP-25 and pSNAP-25 T138 were differently modified by nerve-induced muscle contraction. SNAP-25 remained stable through in both conditions (Ctrl versus ES and ES versus ES + C). Presynaptic stimulation did not affect pSNAP-25 T138, while nerve-induced muscle contraction elevated its protein levels, indicating that pSNAP-25 T138 is upregulated by muscle contraction. On the other hand, Synapsin-1 and pSynapsin-1 S9 responded equally to all the stimulation conditions (Fig. 6B): presynaptic stimulus (Ctrl versus ES) enhanced their levels, while postsynaptic activity (ES versus ES + C) decreased levels of Synapsin-1 and pSynapsin-1 S9.

Moreover, to ensure that neuromuscular activity action on SNAP-25 and Synapsin-1 phosphorylation is conveyed through PKA, we studied the effect of neuromuscular activity after PKA blockade with H-89 (Fig. 6C, D). H-89 is a cell-permeable, potent, and reversible ATP-competitive inhibitor of PKA (*K*_i_ = 48 nM). As expected, PKA downregulation with H-89 abolished the muscle contraction effect on pSNAP-25 (Fig. 6C). This demonstrates that muscle contraction upregulation of pSNAP-25 requires PKA activity. However, after PKA blockade, presynaptic activity was able to maintain pSNAP-25, indicating that PKA is not involved in the endogenous phosphorylation of SNAP-25. We cannot discard that other mechanisms confluence on SNAP-25 phosphorylation acting together with PKA. Finally, the PKA blockade did not affect the total amount of SNAP-25 in any condition of stimulation.

Figure 6D shows that PKA blockade decreases pSynapsin-1 levels during either presynaptic activity or presynaptic activity with contraction, indicating the involvement of PKA activity in phosphorylating Synapsin-1 in both conditions. Presynaptic activity promotes Synapsin-1 phosphorylation using PKA activity, while, on the contrary, nerve-induced muscle contraction downregulates pSynapsin-1 levels by downregulating PKA activity (maybe decreasing Cβ subunit). However, we cannot discard an additive effect of muscle contraction and H-89 on pSynapsin-1 levels. Results also show that the increase in Synapsin-1 levels induced by presynaptic activity is not dependent on PKA, while it is in the muscle contraction condition. In conclusion, PKA is responsible for the increase of pSynapsin-1 induced by presynaptic activity but not for the increase in total Synapsin-1. Muscle contraction induces downregulation of both, Synapsin-1 and pSynapsin-1, and PKA opposes these downregulations. It is possible that the decrease in pSynapsin-1 is a direct consequence of the decrease in Synapsin-1 total protein. Figure 6E shows a graphical summary of the findings.

## Discussion

The interaction between the nervous system and muscles is fundamental for our health. Besides the classic signal from the brain to the muscle, the retrograde signals from the muscle to the brain are just as important. The NMJ is at the center of this bidirectional communication and uses several pathways to regulate neurotransmission in the short term and maintain synaptic function in the long term. To date, the NMJ bidirectional communication has mainly been associated with the BDNF/TrkB signaling and protein kinase C (PKC) pathway [38–40]. Muscle contraction enhances BDNF levels in vitro and in vivo [54, 55], and this BDNF/TrkB signaling retrogradely regulates presynaptic isoforms of PKC and the neurotransmission machinery [38, 39, 55]. However, PKA is also an excellent candidate for its many actions. In the short term, it constitutively enhances ACh release at the NMJ [36, 37] and modulates neural ion channel conductivity [56]. In the long term, PKA modulates acetylcholine receptor stability at the NMJ [57], changes the mRNA translation rate of a small subset of synaptic proteins [58, 59], and participates in NMJ regeneration [60]. Also, PKA and PKC collaborate in the regulation of ACh release at the NMJ [37, 41], and PKA has been linked with retrograde signaling during NMJ development [61]. Here, we demonstrate that presynaptic stimulus and its induced muscle contraction differentially regulate the PKA subunit dynamics to be catalytic active at the NMJ and to phosphorylate molecules of the release machinery (SNAP-25 and Synapsin-1). Our results show that synaptic Cβ subunit regulated by RIIβ or RIIα subunits control activity-dependent phosphorylation of SNAP-25 and Synapsin-1, respectively. Muscle contraction retrogradely downregulates presynaptic activity-induced pSynapsin-1 while enhancing pSNAP-25T138. Both actions could coordinately contribute to decreasing the neurotransmitter release at the NMJ.

### Pre- and postsynaptic activities differently regulate PKA subunit level and dynamics in skeletal muscle

The arrival of the nerve impulse at the presynaptic terminal of the NMJ triggers the release of ACh and results in muscle contraction. Several molecular pathways carefully regulate neurotransmission, including PKA signaling [1–7, 36, 62]. This kinase promotes neurotransmitter release via the phosphorylation of the release machinery [2, 20]. It is important to know how PKA is regulated by synaptic activity and whether it can be retrogradely regulated by the resulting muscle contraction. This will add evidence to understanding the bidirectional communication occurring at the NMJ that contributes to maintaining the synapse functional and healthy and that has been associated with muscle contraction [38–40].

The activity of the C and R subunits of PKA, besides cAMP availability, is controlled by transcriptional or posttranscriptional changes in the synthesis or degradation [24–26]. At NMJs, there is presence of presynaptic and postsynaptic pools of mRNA that fuel the production of synaptic proteins and regulate their levels to support vesicle cycling. Terminal axons can receive and gather a pool of mRNA from the surrounding glial cells, accelerating their adaptation without depending on the slow response from the soma (reviewed in Giuditta et al. [63]). Soluble proteins from the cytosol can be sequestered to these vesicle reserve pools to support neurotransmission. In NMJs, there is a reserve pool of synaptic proteins, including Synapsin-1, that exists in synaptic vesicles and acts as a buffer [64]. When we detect a protein in whole-cell lysates, these samples include the pool of protein in both cytosol and membrane. Therefore, the observed changes occur in the total protein amount, and one must consider variations of either expression or degradation.

Here we found that PKA catalytic subunits were differently affected by synaptic activity. PKA Cα levels did not vary in any treatment, so it could be considered as constitutive in the skeletal muscle and not regulated by synaptic activity. However, PKA Cβ increased in presynaptic stimulus conditions and decreased during nerve-induced muscle contraction. The decrease of Cβ levels during muscle contraction (ES versus ES + C) was induced following proteasomal activity and activity-induced degradation of the subunit. Therefore, it seems that the nerve-induced PKA Cβ levels are retrogradely downregulated by the contraction effect. This retrograde regulation might be due to BDNF/TrkB, as our preliminary data show that the effects of presynaptic stimulus and muscle contraction on Cβ can be enhanced with exogenous BDNF treatment.

Furthermore, the contraction-induced downregulation of PKA Cβ, detected at the membrane and cytosolic fractions, could be related with the M_2_ muscarinic ACh receptors (mAChRs), which downregulate ACh release at the NMJ [36]. The M_2_ muscarinic receptors are G-protein-coupled receptors (GPCRs) that use G proteins to inhibit adenylyl cyclase and PKA. Furthermore, PKA Cβ is inhibited by M_2_ mAChR in basal conditions at the NMJ [20] like contraction does, and this relationship could explain the current PKA Cβ results. Coincident with this interpretation, preliminary results show that muscle contraction downregulates M_2_ mAChR while presynaptic stimulus enhances it. Alternatively, we cannot discard an activity-induced degradation of PKA Cβ [65] caused by the nerve-induced muscle contraction to explain the decrease in the levels of Cβ subunit, as the downregulation occurs in both the cytosolic and membrane fractions and occurs in parallel with increasing pSNAP-25 T138 levels (see “Discussion”). This mechanism would be associated with the modulatory action of the regulatory subunits and proteasomal activity that leads to the activity-induced degradation of the subunit PKA Cβ.

PKA regulatory subunits are directly involved in the regulation of the activity of the catalytic ones. Therefore, the study of their regulation is crucial to understanding the PKA signaling. Here we show that PKA regulatory subunits were regulated differently by synaptic activity. Thus, RIα seems to be regulated only by the postsynaptic contraction, suggesting its involvement in a contraction-dependent postsynaptic mechanism. RIβ is regulated by a presynaptic stimulus-dependent mechanism. Interestingly, RIIα and RIIβ follow opposed dynamics. RIIα decreased in Ctrl versus ES but increased in ES versus ES + C condition. RIIβ, however, increased by presynaptic activity (Ctrl versus ES) but decreased by nerve-induced muscle contraction (ES versus ES + C). Therefore, RIIα downregulation in presynaptic stimulus condition would permit catalytic subunits (maybe Cβ) to phosphorylate targets involved in ACh secretion. On the other hand, during the muscle contraction condition, RIIα would act oppositely by binding to catalytic subunits (maybe Cβ) and inhibiting their activity and ACh liberation. This is in concordance with the dynamic of RIIα in the membrane and explains the effect of presynaptic and muscle contraction activities over pSynapsin-1 levels (see below). On the contrary, presynaptic stimulus condition would increase RIIβ and prevent catalytic subunits (maybe Cβ) from phosphorylating targets, whereas muscle contraction condition would decrease RIIβ and allow the activity of catalytic subunits. This is in concordance with the dynamic of RIIβ in the cytosol and would be closely related with SNAP-25 T138 phosphorylation as this phosphorylation is promoted in the opposite way by pre- and postsynaptic activities (see below). In summary, each PKA regulatory subunit has its own precisely adjusted regulation mechanism. Although the regulation of RIα and RIβ remains unclear, RIIα and RIIβ are oppositely regulated by pre- and postsynaptic activities and can inversely regulate the activity of Cβ subunit. Together, the present results indicate that several activity-dependent mechanisms can balance the final catalytic activity of Cβ at the NMJ.

### Postsynaptic contraction modulates AKAP150

Targeting of PKA to specific intracellular sites is achieved by AKAPs [66, 67]. Aside from AKAPs, regulatory subunit translocation to the membrane could also be related to Gαi/0 association [68]. We previously showed that changes in AKAP150 protein expression are associated with the recruitment of PKA regulatory subunits to the membrane [20], so free catalytic subunits are able to phosphorylate their substrates involved in ACh transmission. Here, we show that nerve-induced muscle contraction enhances AKAP150 at the NMJ, suggesting that AKAP150 would recruit PKA regulatory subunits to the membrane, so cytosolic catalytic subunits would be able to phosphorylate their substrates. The dynamic of RIIβ in the cytosol (decrease) coincides with this regulation of AKAP150 during contraction. However, neither RIIα, RIα, nor RIβ seems regulated by AKAP150 in the activity conditions. The effects on pSNAP-25 T138 under contraction confirm this mechanism of AKAP150 and RIIβ (see below).

### Pre- and postsynaptic activities modulate Synapsin-1 and SNAP-25 PKA phosphorylation

We observed the phosphorylation of targets to understand the regulation of kinase activity. Some PKA targets involved in transmitter release are *N*-ethylmaleimide-sensitive factor attachment protein alpha (α-SNAP) [69], cysteine string protein (CSP) [70], Synapsin-1 [71], snapin [72], syntaphilin [73], rabphilin [74], Rab3 interacting protein 1α (RIM1α) [75], and SNAP-25 [33, 34]. SNAP-25, together with synaptobrevin and syntaxin, forms the SNARE core complex, which is involved in vesicle docking, priming, and triggering fast exocytosis [76, 77]. SNAP-25 is a presynaptic target of PKA on its T138 [34], and this phosphorylation controls the size of the releasable vesicle pools and occurs in response to synaptic activity [30]. Nonphosphorylated SNAP-25 strongly interacts with Syntaxin-1 until phosphorylated [78]. Once phosphorylated, SNAP-25 switches to promote vesicle recycling and recruitment [2, 30, 79].

Here we found that SNAP-25 and pSNAP-25 T138 were differently regulated by synaptic activity. Coincident with our previous study at the NMJ [39], SNAP-25 remained unaltered in all conditions, indicating that its total levels are not regulated by synaptic activity. Meanwhile, pSNAP-25 T138 was stable in presynaptic stimulus condition and increased during nerve-induced muscle contraction. This is mediated through PKA, because the effect of muscle contraction is abolished by a previous incubation with H-89. As mentioned earlier, the contraction-induced pSNAP-25 T138 increase could be related with the upregulation of AKAP150 in the same conditions: AKAP would recruit enough PKA regulatory subunits to allow PKA catalytic subunits to phosphorylate pSNAP-25 T138. When SNAP-25 is phosphorylated at the T138 by PKA, it loses its interaction with syntaxin-1 and the SNARE complex is disassembled. Thus, the increase of pSNAP-25 T138 during contraction would regulate the stability of the SNARE-SM complex and a correct balance of the size of the releasable vesicle pools for future activity bursts.

Recent results indicated that Cβ is enriched in the muscle synaptic areas of the diaphragm while Cα is almost equally distributed between synaptic and extrasynaptic regions [20]. According to this and our current results, Cβ and RIIβ subunits could be good candidates to regulate PKA-induced SNAP-25 phosphorylation. We detected a decrease of Cβ (induced by activity-dependent degradation) coincident with a decrease in RIIβ in the cytosol (possibly promoted by AKAP150). Nevertheless, it is interesting to note that pSNAP-25 T138 did not increase in presynaptic stimulus—when Cβ is augmented—but raised in muscle contraction—when Cβ diminished. This indicates that the regulatory mechanism is stronger during contraction than during presynaptic stimulus. Thus, pSNAP-25 T138 phosphorylation appears to be more related to AKAP150 and RIIβ protein levels than to those of Cβ. Additionally, we should not discard the possibility that another kinase upregulates pSNAP-25 T138 during contraction beyond the activity-dependent Cβ. Furthermore, SNAP-25 is also phosphorylated by PKC in S187 [34]. At the NMJ, pSNAP-25 S187 increases by presynaptic stimulus and decreases by muscle contraction [39], different variations from those of pSNAP-25 T138. These differences suggest that a specific ratio in SNAP-25 phosphorylations by PKC and PKA could guarantee the correct functionality of the molecule in the synaptic vesicle exocytosis. Ser187 phosphorylation of SNAP-25 enhances the recruitment after the releasable vesicle pools have been emptied [30], and this phosphorylating mechanism is induced by synaptic activity [30, 39]. On the other hand, T138 phosphorylation of SNAP-25 by PKA controls the size of the releasable vesicle pools [30].

The results show that phosphorylation of SNAP-25 at T138 is not modified by the presynaptic stimulus, indicating that a change of this phosphorylation is not crucial to the ACh release at the NMJ, unlike the phosphorylation of SNAP-25 at S187 or Munc18-1 [38, 39], among others. This is in line with biochemical studies showing that PKA-dependent phosphorylation of SNAP-25 does not regulate ternary SNARE complex assembly and that T138 is not an essential residue for complex formation. However, it may serve to modulate SNARE complex function through other proteins, including synaptotagmin I [33]. This is in concordance with the endogenous PKA activity at the NMJ on ACh release [36]. In addition, we previously found that, in basal conditions, the inhibition of mAChR-M_1_, which decreases ACh release [36], does not affect SNAP-25 T138 phosphorylation [20]. This and the present results suggest a PKA-independent phosphorylation on SNAP-25 T138 to promote presynaptic activity-induced ACh release. The stability of Cα and the lack of regulation of AKAP150 could be related with this PKA endogenous mechanism that is not regulated by activity. However, it is very stimulating to hypothesize that the balance between the opposite regulations of RIIβ and RIIα on Cβ activity in presynaptic stimulus condition (which are opposite from the contraction regulation) controls the stability of SNAP-25 T138 phosphorylation.

Recently, it has been shown that mAChR-M_2_ inhibition in basal condition (which increases the ACh release) induces an important increase in SNAP-25 T138 phosphorylation, which can be abolished by a previous incubation with H-89. This finding demonstrates that mAChR-M_2_ inhibits the phosphorylation of SNAP-25 at T138, which is modulated by PKA activity [20]. These results demonstrate a relation between mAChR-M_2_, Cβ subunit, and SNAP-25 T138 phosphorylation. Here we found that the contraction condition has the same effect as the mAChR-M_2_ inhibition on RIIβ subunit, AKAP150, and SNAP-25 T138 phosphorylation. Thus, we could suggest that contraction downregulates mAChR-M_2_ and therefore the final phosphorylation of SNAP-25 T138 will increase, as we found here. Further experiments are needed to confirm this hypothesis.

Interestingly, the activity-induced dynamic of Synapsin-1 phosphorylation is different from that of SNAP-25 T138 phosphorylation. pSynapsin-1 level increases during presynaptic stimulation, and nerve-induced muscle contraction downregulates it. Both conditions downregulated pSynapsin-1 level during previous incubation with H-89, demonstrating that the Synapsin-1 phosphorylation involves PKA activity at the NMJ. Levels of pSynapsin-1 and Cβ subunit change accordingly, suggesting a direct relationship. Furthermore, the results also show that RIIα may contribute to the regulation of Cβ subunit to phosphorylate Synapsin-1. Synapsin-1 phosphorylation induced by presynaptic activity would contribute to ACh release as synapsins regulate vesicle clustering and therefore neurotransmitter release [71, 80–88]. In particular, Synapsin-1 is responsible for the tethering of synaptic vesicles to the actin cytoskeleton contributing to the synaptic vesicles trafficking that helps to maintain the reserve pool of vesicles close to the active zone [89]. In accordance with this, knockout of Synapsin-1 in mice resulted in disruption of synaptic vesicles clustering and subsequent decrease of the glutamate release [80]. Synapsin-1 phosphorylation in the central nervous system promotes vesicle mobilization into the releasable pool [90], which allows Synapsin-1 to carry out its function in the nerve terminal liberating synaptic vesicles from actin [91]. Muscle contraction-induced downregulation of pSynapsin-1 would attach synaptic vesicles to the actin cytoskeleton, preventing their clustering to the active zones. This muscular feedback could be in relation with the extended control of the neuromuscular ACh release by the negative action of the M_2_ muscarinic AChR and A1 adenosine receptor [92–94]. However, phosphorylation of Synapsin-1 at Ser9 is regulated by PKA and CAMKI/IV [95] Further experiments are necessary to know whether CAMKI/IV activity is involved in the synaptic activity effect on pSynapsin-1.

Here we found that the total protein of Synapsin-1 changes in parallel to pSynapsin-1 in response to pre- and postsynaptic activities. A previous incubation with H-89 affects Synapsin-1 and pSynapsin-1 levels differently, suggesting different PKA involvement. H-89 preincubation in the presynaptic activity conditions did not change Synapsin-1 levels. Thus, PKA is not involved in the increase of Synapsin-1 during presynaptic stimulus. However, Synapsin-1 levels decrease when H-89 is preincubated during contraction, indicating that PKA activity contributes to maintaining Synapsin-1 levels in the nerve terminal, in opposition to the contraction effect. In addition, contraction could downregulate PKA levels, resulting in the Cβ subunit decrease observed here. The results indicate that contraction triggers the PKA positive regulation over Synapsin-1. Regarding Synapsin-1 phosphorylation, H-89 preincubation reduces pSynapsin-1 levels during presynaptic activity and muscle contraction. Thus, PKA activity phosphorylates Synapsin-1 during both activity conditions. Because muscle contraction induces downregulation of both Synapsin-1 and pSynapsin-1 and PKA opposes both downregulations (ratio pSynapsin-1/Synapsin-1 remains unchanged), the decrease in pSynapsin-1 could be a direct consequence of the decrease in total protein.

Several subunits of PKA have been located at the NMJ [20–23]. In particular, Cβ subunit is located at the nerve terminal but also at the postsynaptic muscle cell and at the perisynaptic glial cell [20], suggesting that PKA signaling occurs in all cells. Here we have shown how changes in regulatory and Cβ subunits occur in parallel to PKA targets located at the nerve terminal of the NMJ, indicating that the proposed signaling is presynaptic. Previously, we determined that SNAP-25 is located at the nerve terminal [20, 38]. Here we confirm this and localize Synapsin-1 in the same synaptic area as Syntaxin. Syntaxin is a presynaptic marker of the nerve terminal of the NMJ as it is a transmembrane protein on the plasma membrane that is part of the SNARE complex. This presynaptic location is concordant with previous studies in different types of cells [96] and with the known synaptic function of Synapsin-1 in the synaptic vesicle cycle. Moreover, although Synapsin-1 was located in the same synaptic area as Syntaxin, they are not completely colocalized, indicating that both molecules have a specific distribution in the nerve terminal related to their function.

As there are postsynaptic PKA substrates, we cannot discard that the activity-dependent effect of PKA on these targets could be involved in the retrograde signaling pathway here described. Indeed, it is well known that a balance of PKA and PKC regulates the stability of AChRs at the NMJ, both during development [97–101] and in the adult [23, 46, 102]. Moreover, synaptic activity has a role in regulating the receptor density/turnover rate at the NMJ [103] contributing to the synaptic plasticity of the neuromuscular system [57].

The results provide evidence that nerve terminals need both pre- and postsynaptic activities to modulate SNAP-25 and Synapsin-1 phosphorylation and ensure an accurate neurotransmission process. It is known that muscle contraction further enhances the nerve-induced BDNF levels [54, 55], and that the BDNF/TrkB downstream signaling regulates retrogradely presynaptic PKC isoforms controlling neurotransmission machinery [38, 39, 55]. Furthermore, because PKC and PKA collaborate in the regulation of the ACh release at the NMJ [37, 41], a BDNF/TrkB-dependent retrograde regulation on PKA is strongly implied. Preliminary unpublished results drive us to think that TrkB signaling controls the PKA activity at the NMJ.

## Conclusions

**Fig. 7:**
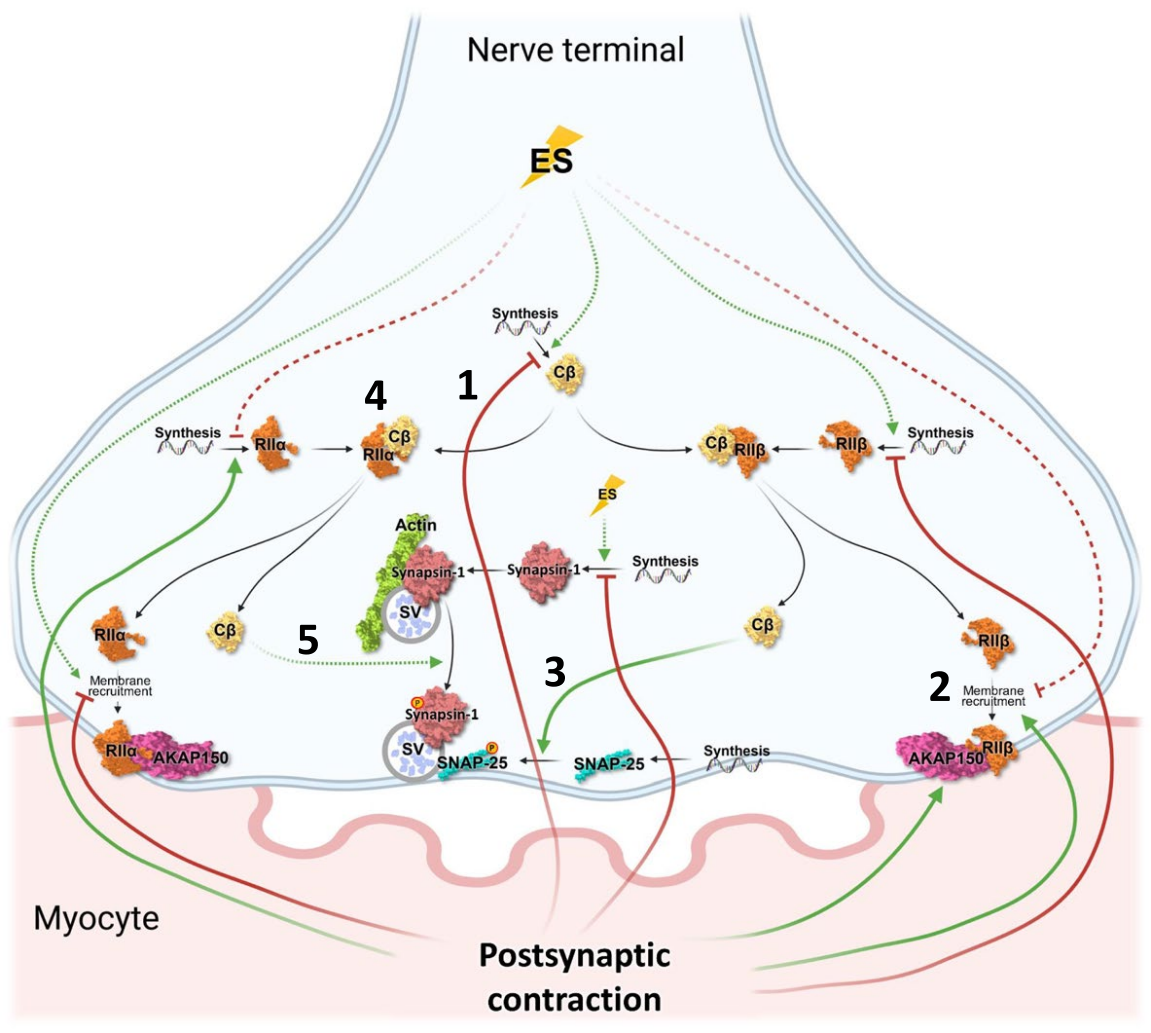
Summary of PKA subunits regulation at the NMJ during nerve-induced muscle contraction. Model of the PKA regulation resulted from this study during nerve-induced muscle contraction. Muscle contraction opposes the presynaptic stimulation in many steps of the signaling. (1) It decreases Cβ levels maybe as a result of the activity-induced degradation of the subunit because (2) the contraction-induced upregulation of AKAP150 is capable of recruiting enough RIIβ regulatory subunits (3) to permit Cβ catalytic subunits to increase their activity enhancing pSNAP-25 T138 phosphorylation. In addition, (4) RIIα opposes to this regulation binding to Cβ to inhibit its activity contributing to balance (5) the catalytical activity of Cβ on pSynapsin-1 S9. These results indicate that bidirectional communication and balance between pre- and postsynaptic activity provide the optimal conditions to maintain the stable neurotransmission of ACh

It is known that PKA activity enhances ACh release at the NMJ. In the present work, we determined the activity-dependent dynamics of PKA subunits at the NMJ, which are related with phosphorylation of targets involved in synaptic vesicle exocytosis (SNAP-25 and Synapsin-1). Figure 7 shows a summary of the nerve-induced muscle contraction effects over PKA subunits and AKAP150 and their interactions to regulate ACh release through the phosphorylation of SNAP-25 T138 and Synapsin-1 S9. Our results show that nerve-induced muscle contraction decreases Cβ levels as a result of the activity-induced degradation of the subunit because the contraction-induced upregulation of AKAP150 is capable of recruiting enough RIIβ regulatory subunits to permit Cβ catalytic subunits to increase their activity, enhancing pSNAP-25 T138 phosphorylation. Additionally, the muscle contraction-induced increase in RIIα may retain Cβ subunits, thus decreasing the nerve-induced Synapsin-1 phosphorylation. Both retrograde regulations could be a feedback mechanism to reduce the nerve-induced ACh release, once the postsynaptic cell contracts. Together, these results suggest that neuromuscular synaptic activity performed by the neuron and the myocyte regulates accurately the PKA catalytic and regulatory subunits to maintain an optimal ACh transmission at the NMJ.

The results provide a molecular mechanism of the bidirectional communication between nerve terminals and muscle cells that contribute to balancing the optimal process of ACh release, which could be important to provide molecules as a therapy for neuromuscular diseases in which the neuromuscular crosstalk is impaired.

## Supplementary Information

**Additional file 1.** Original blots. The western blotting pictures were cropped from the original pictures with different conditions but not modified. Each dot in the bars of the graphs is representing the mean result of one animal.

## Data Availability

We think that our data are not appropriate for the available repository database in neuroscience.

## Abbreviations

ACh: Acetylcholine
AChE: Acetylcholinesterase
AChR: Acetylcholine receptors
AKAP: A-kinase anchoring proteins
AP: Action potential
ATP: Adenosine triphosphate
BDNF: Brain-derived neurotrophic factor
BSA: Bovine serum albumin
C: Catalytic
Ca^2+^: Calcium ion
CAMK: CAM kinase
cAMP: Cyclic adenosine monophosphate
DMSO: Dimethyl sulfoxide
IHC: Immunohistochemistry
LAL: Levator auris longus
mM: Millimolar
µM: Micromolar
nM: Nanometer
NMJ: Neuromuscular junction
NT: Neurotransmitter
PBS: Phosphate-buffered saline
PKA: Protein kinase A
PKC: Protein kinase C
SBL: Reduced sample buffer loading
SEM: Standard error of the mean
Ser: Serine
SNARE: Soluble NSF attachment protein
SV: Synaptic vesicle
PVDF: Polyvinylidene difluoride
R: Regulatory
T: Threonine
TBE: Tribromoethanol
TBS: Tris-buffered saline
TBST: Tris-buffered saline 0.1% Tween 20
TRICT: Tetramethyl rhodamine isothiocyanate
VSDC: Voltage-dependent sodium channels
*v*/*v*: Volume/volume
α-BTX: α-Bungarotoxin
μl: Microliter (10^−^^6^ l)
μ-CgTx-GIIIB: μ-Conotoxin GIIIB
