# Supplementary material for: Synaptic retrograde regulation of the PKA-induced SNAP-25 and Synapsin-1 phosphorylation
Source: Cell Mol Biol Lett. 2023 Mar 3;28:17. doi: 10.1186/s11658-023-00431-2 (PMC9985302; doi:10.1186/s11658-023-00431-2)

The western blotting pictures were cropped from the original pictures with different conditions but not modified. Each **dot** in the bars of the graphs is representing the **mean result of one animal**.

Here we provide some original blots obtained during the experiments.

[red dashed box] - mark for the bands which were chosen for the publication.

FIGURE 2

Fig 2. C Cα

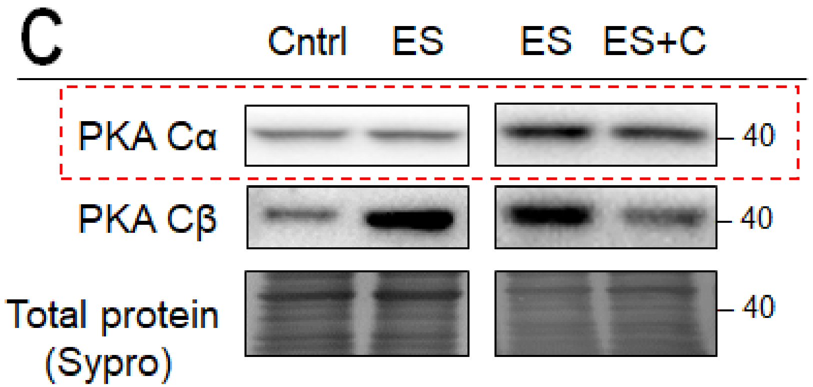

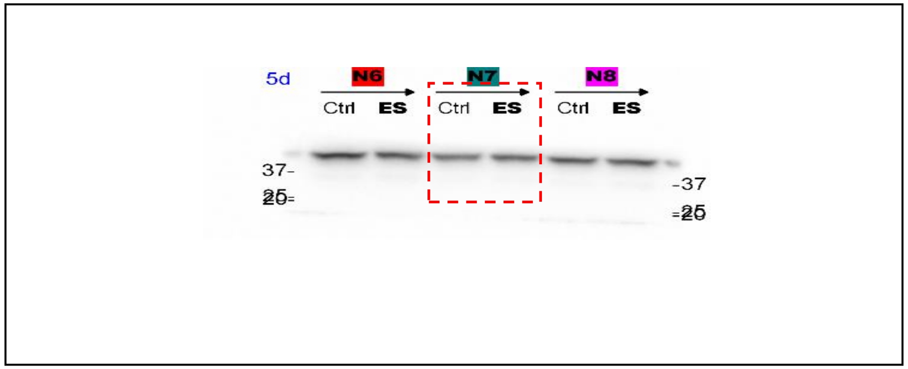

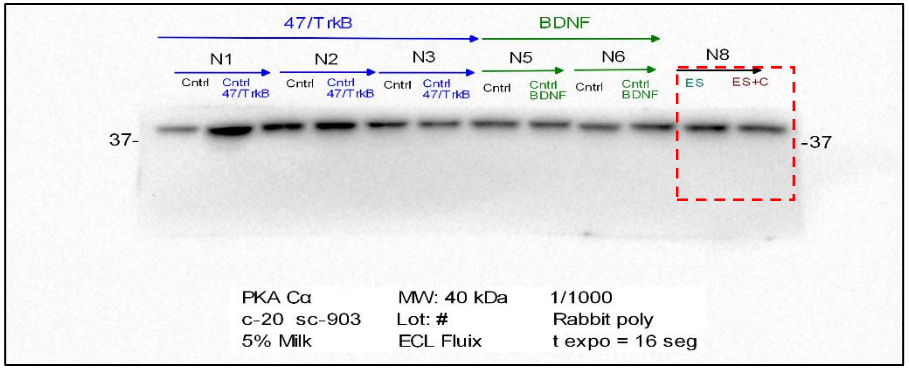

Fig 2. C Cβ

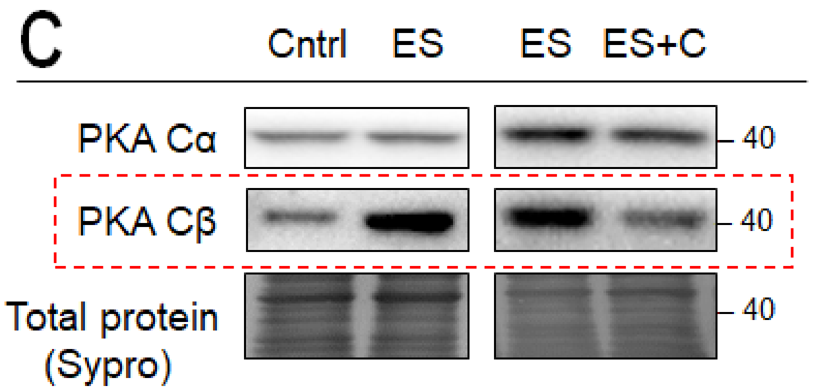
C
Cntrl ES ES ES+C
PKA Cα
PKA Cβ
Total protein (Sypro)
40
40
40

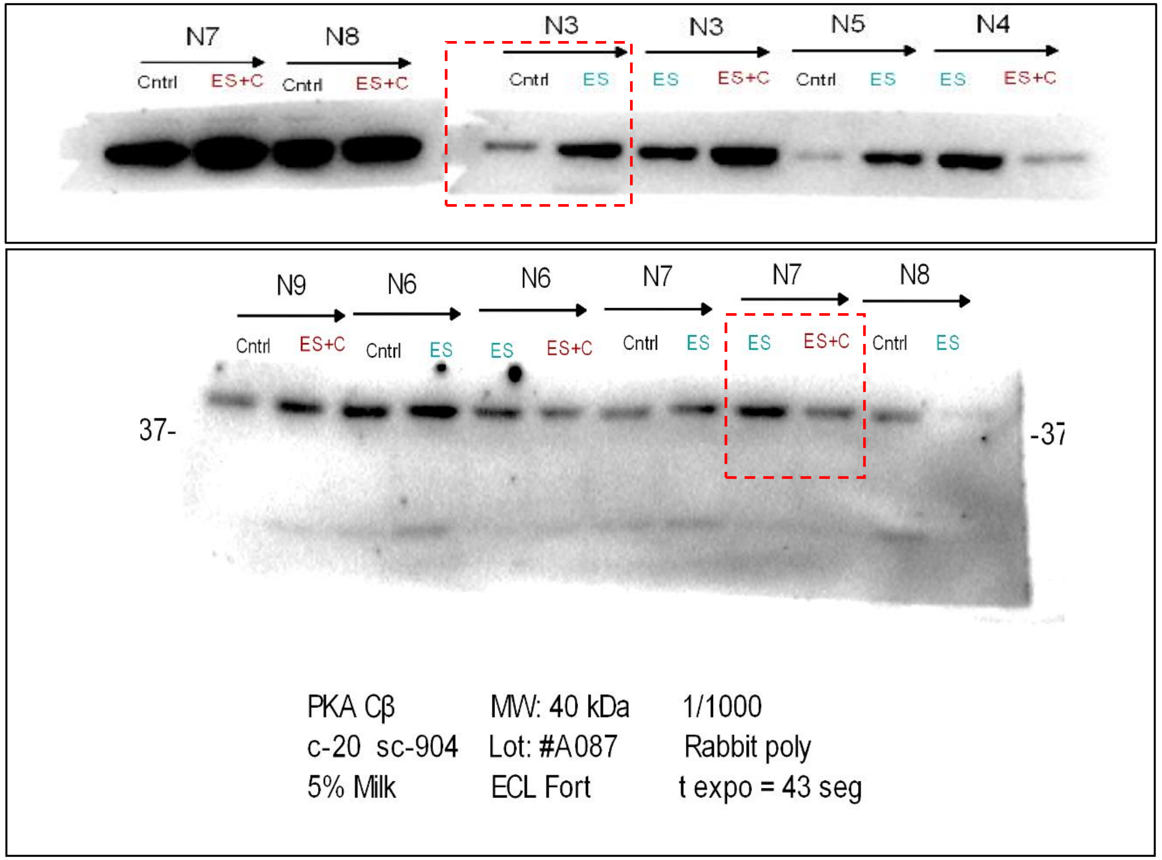
N7 N8 N3 N3 N5 N4
Cntrl ES+C Cntrl ES+C Cntrl ES ES ES+C Cntrl ES ES ES+C
N9 N6 N6 N7 N7 N8
Cntrl ES+C Cntrl ES ES ES+C Cntrl ES ES ES+C Cntrl ES
37-
-37
PKA Cβ
MW: 40 kDa
1/1000
c-20 sc-904
Lot: #A087
Rabbit poly
5% Milk
ECL Fort
t expo = 43 seg

Fig 2. D RIα

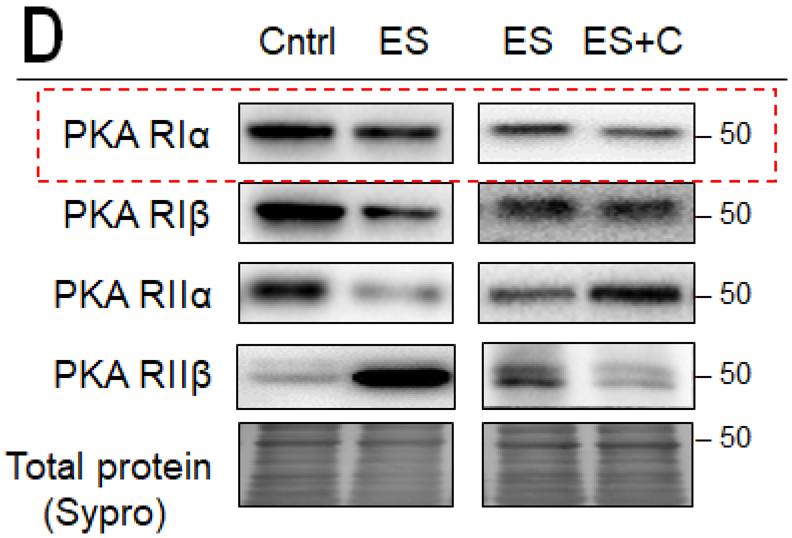
D
Cntrl
ES
ES
ES+C
PKA RIα
PKA RIβ
PKA RIIα
PKA RIIβ
Total protein (Sypro)
50

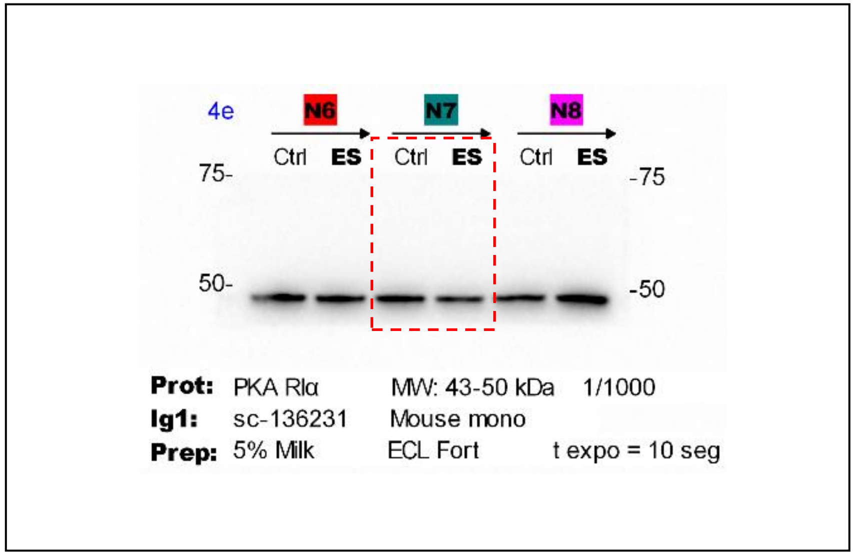
4e
N6
N7
N8
Ctrl
ES
75-
50-
-75
-50
Prot: PKA RIα MW: 43-50 kDa 1/1000
Ig1: sc-136231 Mouse mono
Prep: 5% Milk ECL Fort t expo = 10 seg

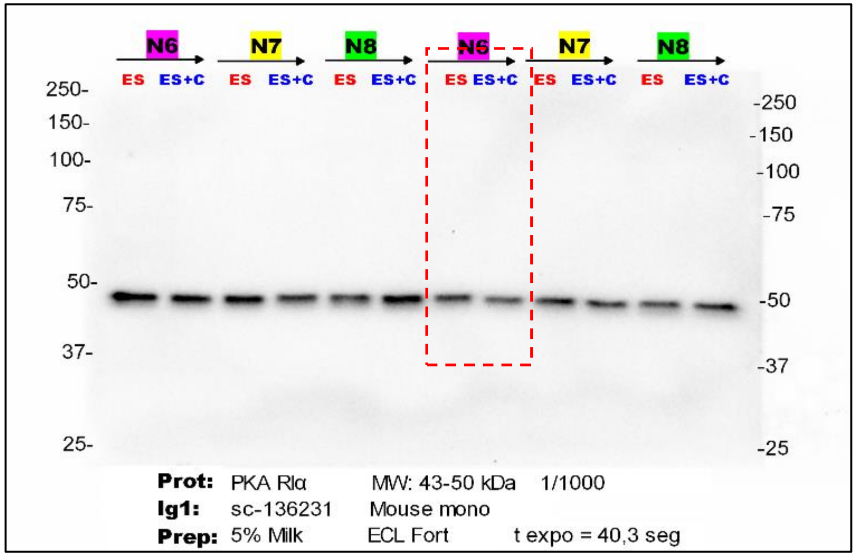
N6
N7
N8
ES
ES+C
250-
150-
100-
75-
50-
37-
25-
-250
-150
-100
-75
-50
-37
-25
Prot: PKA RIα MW: 43-50 kDa 1/1000
Ig1: sc-136231 Mouse mono
Prep: 5% Milk ECL Fort t expo = 40,3 seg

Fig 2. D RIβ

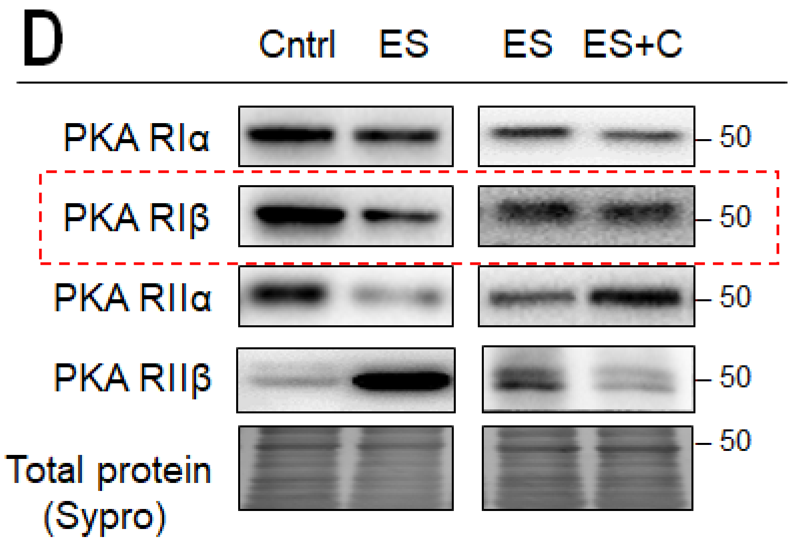

D
Cntrl
ES
ES
ES+C
PKA RIα
PKA RIβ
PKA RIIα
PKA RIIβ
Total protein
(Sypro)
50
50
50
50
50

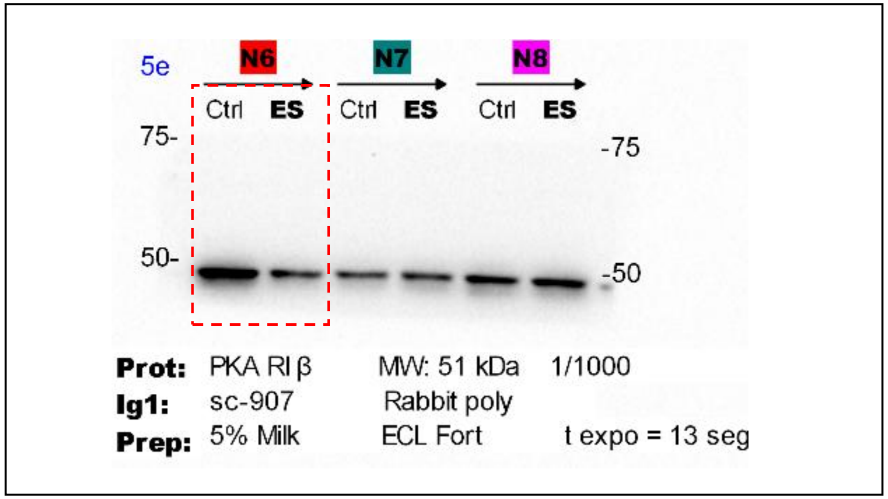

5e
N6
N7
N8
Ctrl
ES
Ctrl
ES
Ctrl
ES
75-
50-
-75
-50
Prot: PKA RI β MW: 51 kDa 1/1000
Ig1: sc-907 Rabbit poly
Prep: 5% Milk ECL Fort t expo = 13 seg

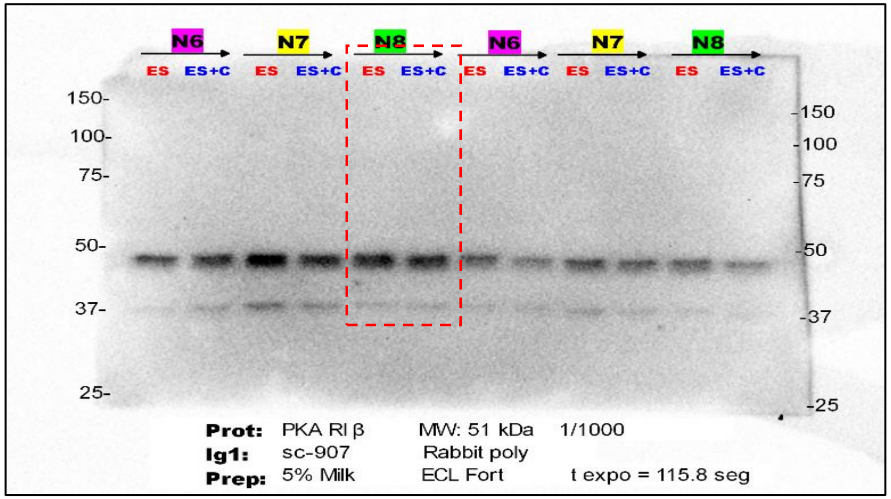

N6
N7
N8
N6
N7
N8
ES
ES+C
ES
ES+C
ES
ES+C
ES
ES+C
ES
ES+C
ES
ES+C
150-
100-
75-
50-
37-
25-
-150
-100
-75
-50
-37
-25
Prot: PKA RI β MW: 51 kDa 1/1000
Ig1: sc-907 Rabbit poly
Prep: 5% Milk ECL Fort t expo = 115.8 seg

Fig 2. D RIIα

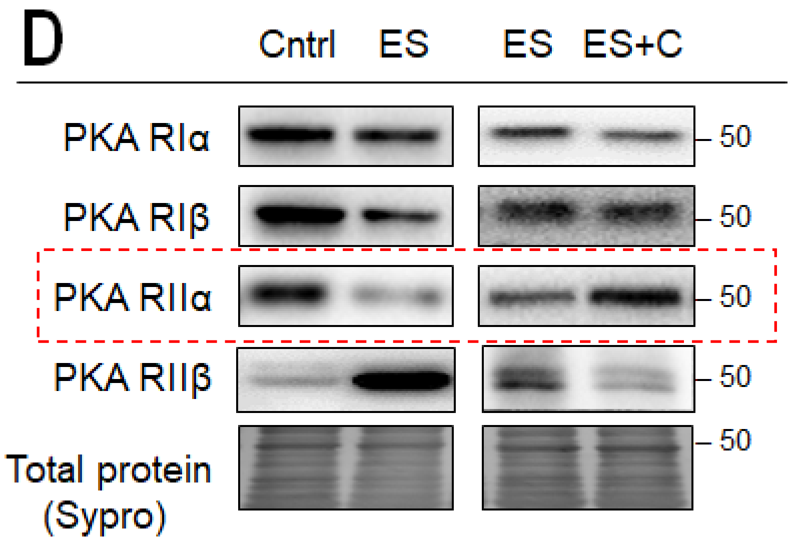
D
Cntrl ES ES ES+C
PKA RIα
PKA RIβ
PKA RIIα
PKA RIIβ
Total protein (Sypro)
50
50
50
50
50

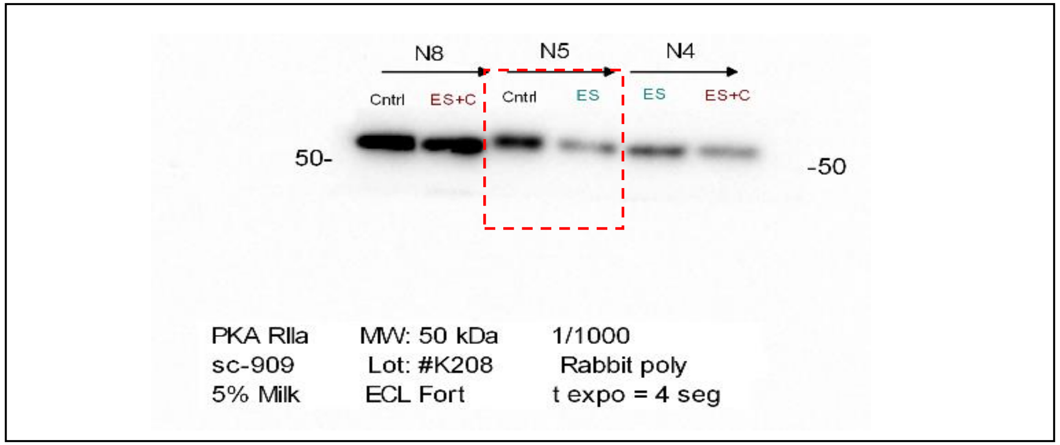
N8 N5 N4
Cntrl ES+C Cntrl ES ES ES+C
50-
-50
PKA RIIa MW: 50 kDa 1/1000
sc-909 Lot: #K208 Rabbit poly
5% Milk ECL Fort t expo = 4 seg

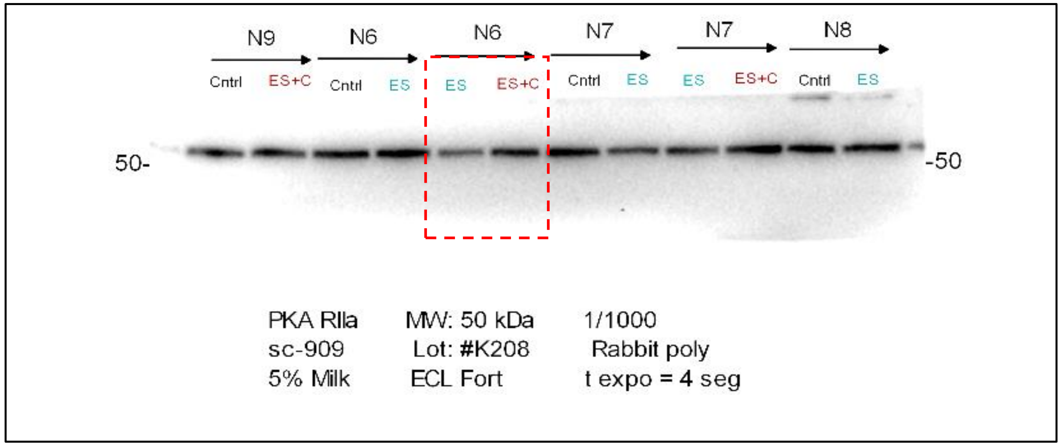
N9 N6 N6 N7 N7 N8
Cntrl ES+C Cntrl ES ES ES+C Cntrl ES ES ES+C Cntrl ES
50-
-50
PKA RIIa MW: 50 kDa 1/1000
sc-909 Lot: #K208 Rabbit poly
5% Milk ECL Fort t expo = 4 seg

Fig 2. D RIIβ

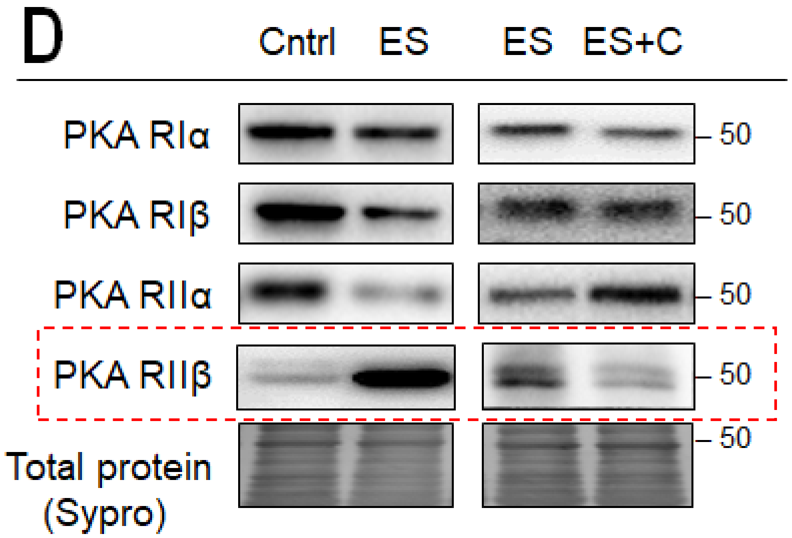
D
Cntrl
ES
ES
ES+C
PKA RIα
PKA RIβ
PKA RIIα
PKA RIIβ
Total protein (Sypro)
50
50
50
50
50

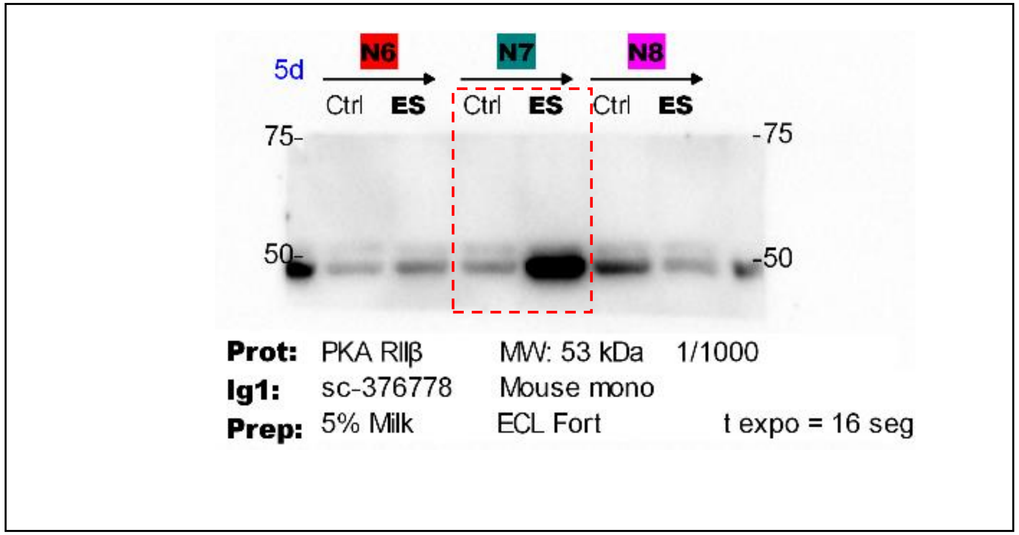
5d
N6
N7
N8
Ctrl
ES
Ctrl
ES
Ctrl
ES
75-
-75
50-
-50
Prot: PKA RIIβ MW: 53 kDa 1/1000
Ig1: sc-376778 Mouse mono
Prep: 5% Milk ECL Fort t expo = 16 seg

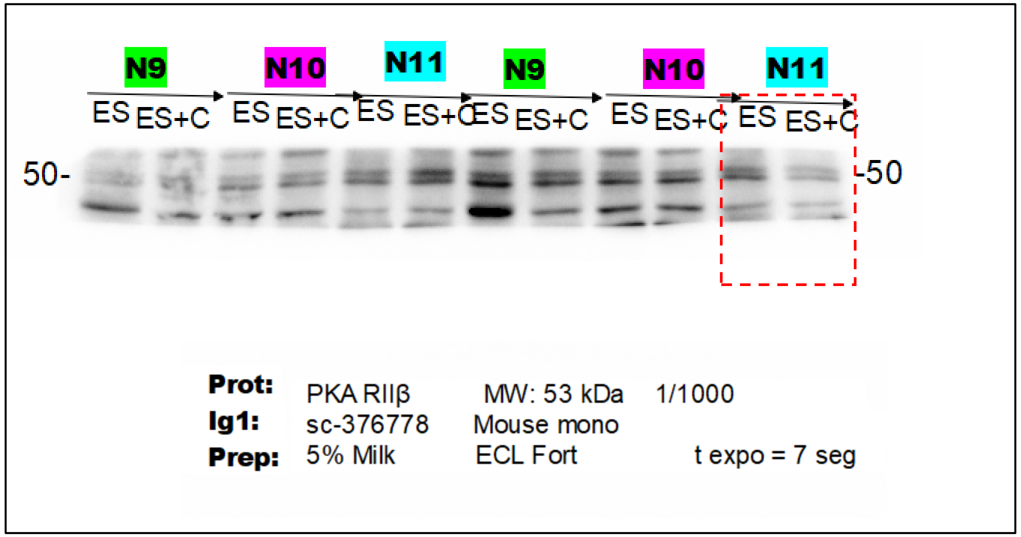
N9
N10
N11
N9
N10
N11
ES
ES+C
ES
ES+C
ES
ES+C
ES
ES+C
ES
ES+C
ES
ES+C
50-
-50
Prot: PKA RIIβ MW: 53 kDa 1/1000
Ig1: sc-376778 Mouse mono
Prep: 5% Milk ECL Fort t expo = 7 seg

Fig 2. E Cβ

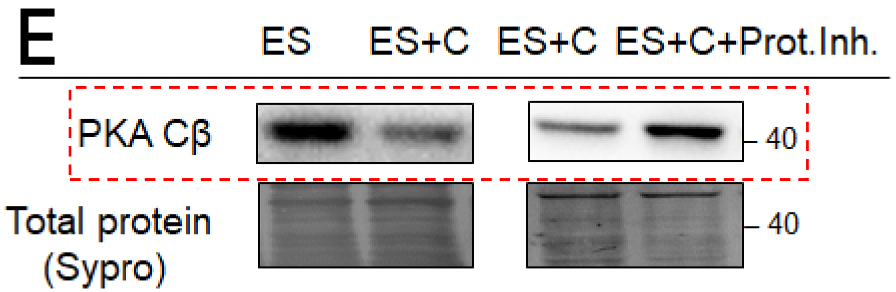
E
ES
ES+C
ES+C
ES+C+Prot.Inh.
PKA Cβ
40
Total protein
(Sypro)
40

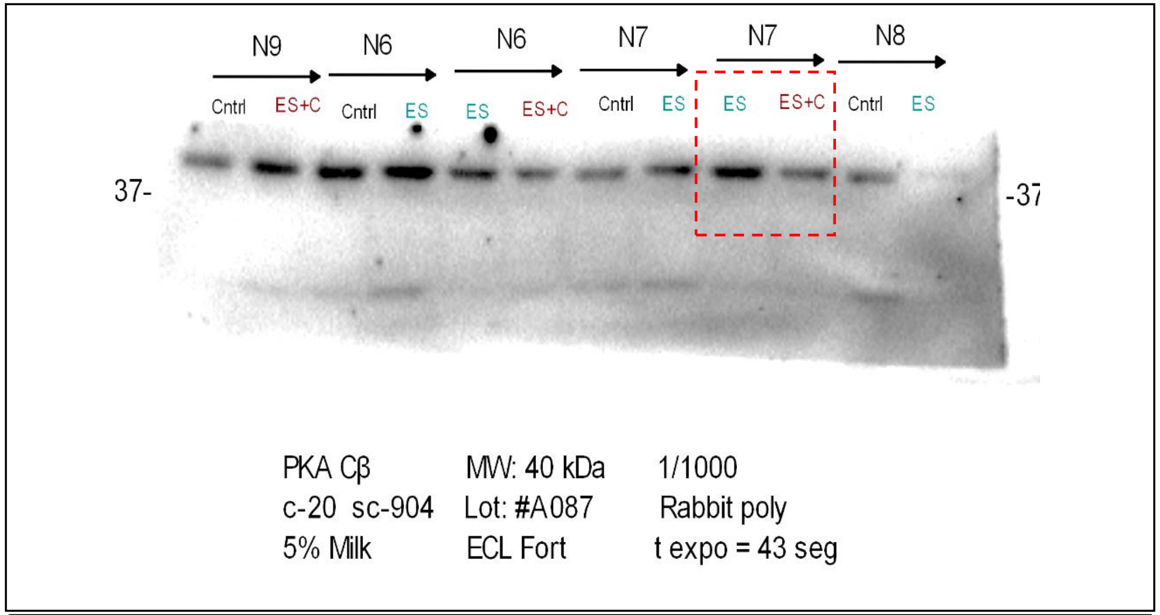
N9
N6
N6
N7
N7
N8
Cntrl
ES+C
Cntrl
ES
ES
ES+C
Cntrl
ES
ES
ES+C
Cntrl
ES
37-
-37
PKA Cβ
MW: 40 kDa
1/1000
c-20 sc-904
Lot: #A087
Rabbit poly
5% Milk
ECL Fort
t expo = 43 seg

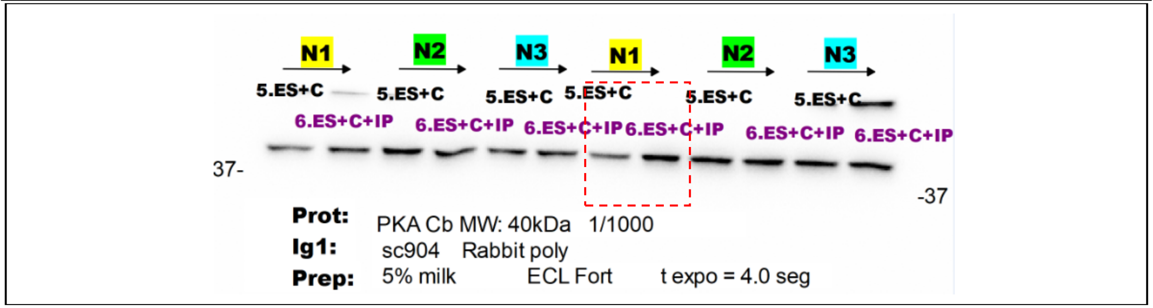
N1
N2
N3
N1
N2
N3
5.ES+C
5.ES+C
5.ES+C
5.ES+C
5.ES+C
5.ES+C
6.ES+C+IP
6.ES+C+IP
6.ES+C+IP
6.ES+C+IP
6.ES+C+IP
6.ES+C+IP
37-
-37
Prot:
PKA Cb MW: 40kDa 1/1000
Ig1:
sc904 Rabbit poly
Prep:
5% milk ECL Fort t expo = 4.0 seg

FIGURE 3

Fig 3. A

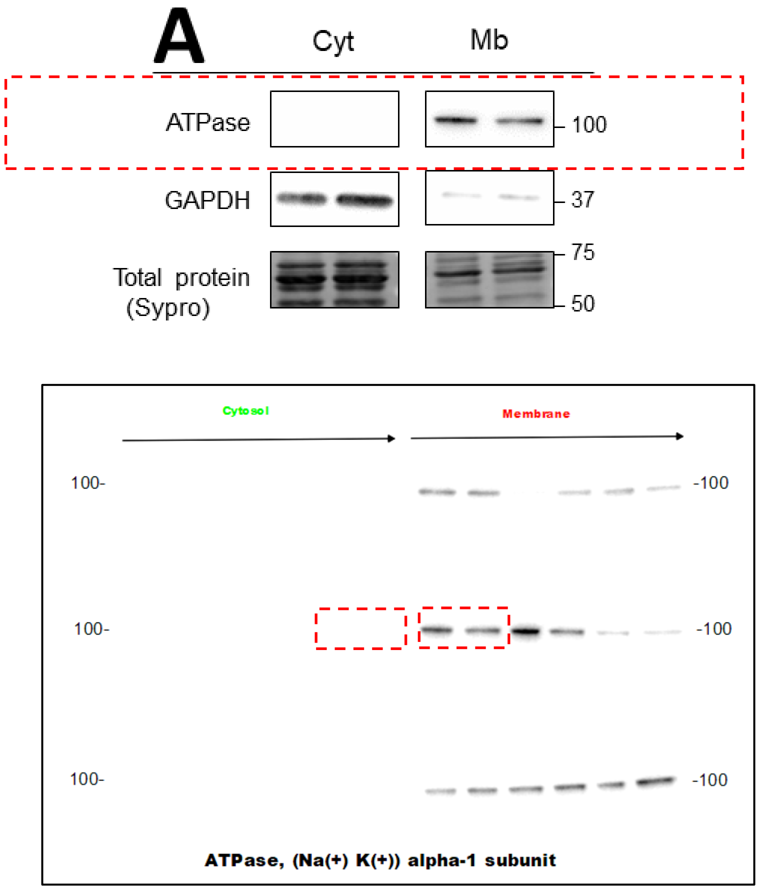

Fig 3. A

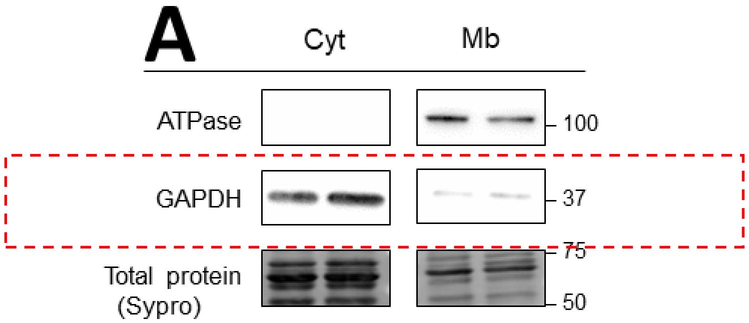
A
Cyt
Mb
ATPase
100
GAPDH
37
Total protein
(Sypro)
75
50

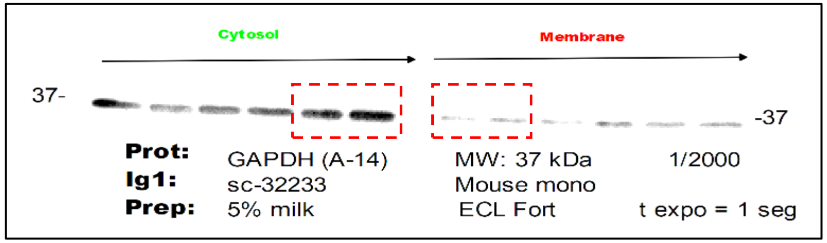
Cytosol
Membrane
37-
-37
Prot: GAPDH (A-14) MW: 37 kDa 1/2000
Ig1: sc-32233 Mouse mono
Prep: 5% milk ECL Fort t expo = 1 seg

Fig 3. C

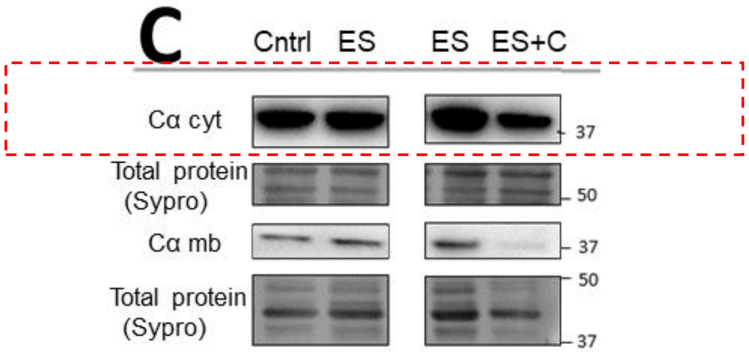
C
Cntrl ES ES ES+C
Cα cyt
Total protein (Sypro)
Cα mb
Total protein (Sypro)
37
50
37
50
37

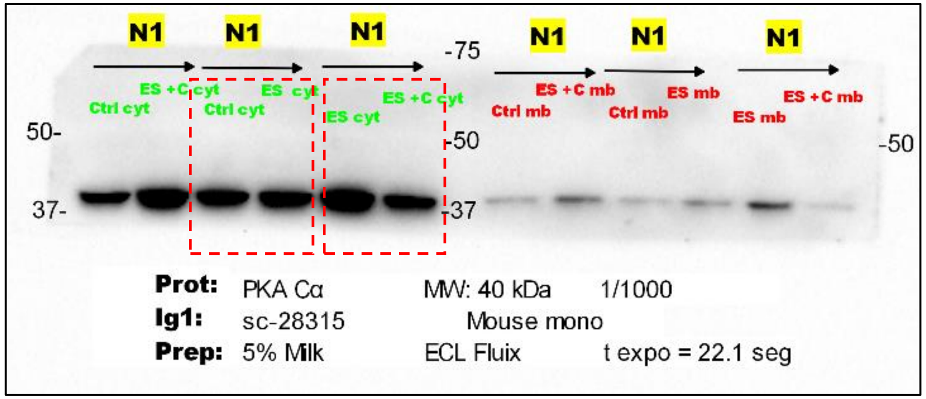
N1
N1
N1
N1
N1
N1
-75
Ctrl cyt
ES +C cyt
Ctrl cyt
ES cyt
ES cyt
ES +C cyt
Ctrl mb
ES +C mb
Ctrl mb
ES mb
ES mb
ES +C mb
50-
-50
-50
37-
-37
Prot: PKA Cα MW: 40 kDa 1/1000
Ig1: sc-28315 Mouse mono
Prep: 5% Milk ECL Fluix t expo = 22.1 seg

Fig 3. C

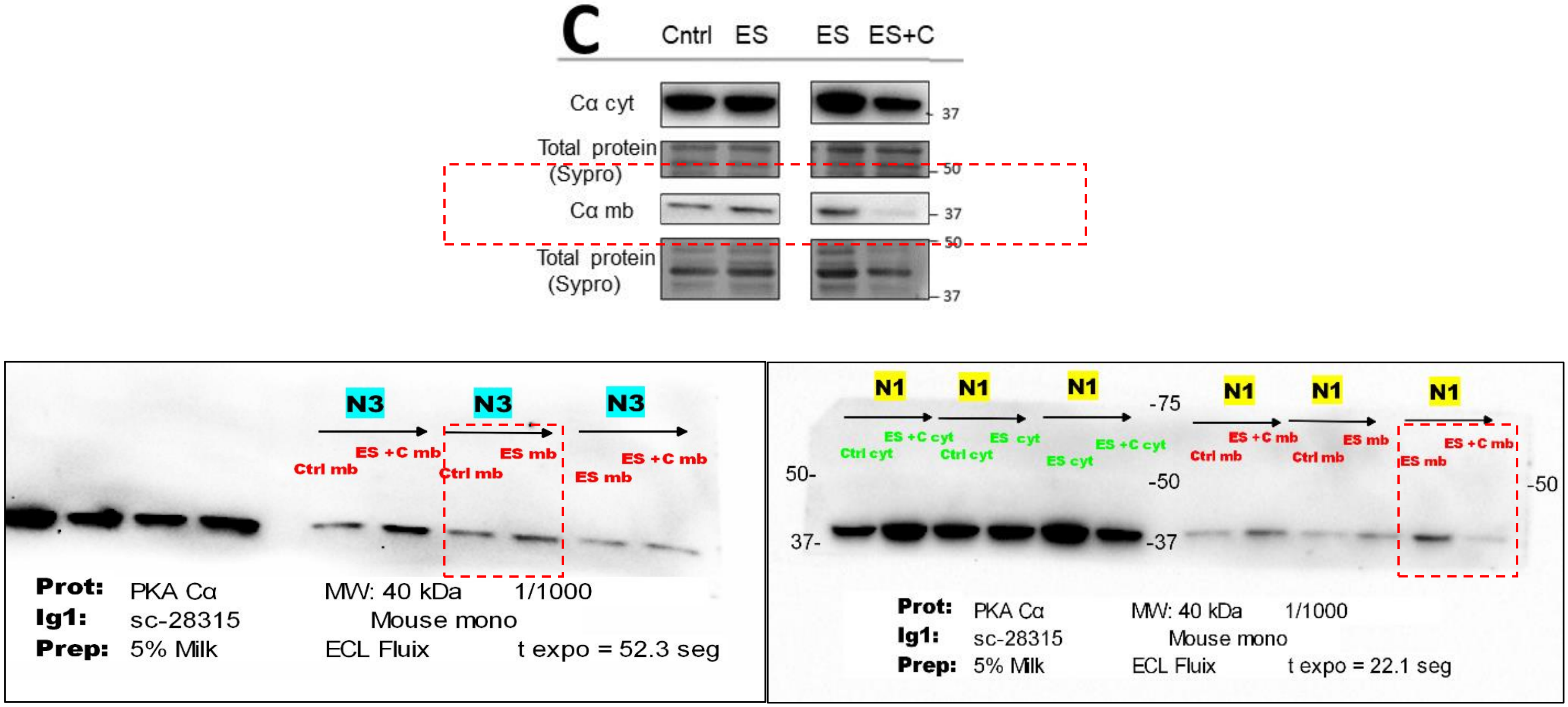

C
Cntrl
ES
ES
ES+C
Cα cyt
37
Total protein (Sypro)
50
Cα mb
37
50
Total protein (Sypro)
37
N3
N3
N3
Ctrl mb
ES +C mb
Ctrl mb
ES mb
ES mb
ES +C mb
Prot: PKA Cα
MW: 40 kDa
1/1000
Ig1: sc-28315
Mouse mono
Prep: 5% Milk
ECL Fluix
t expo = 52.3 seg
N1
N1
N1
N1
N1
N1
-75
Ctrl cyt
ES +C cyt
Ctrl cyt
ES cyt
ES cyt
ES +C cyt
Ctrl mb
ES +C mb
Ctrl mb
ES mb
ES mb
ES +C mb
50-
-50
-50
37-
-37
Prot: PKA Cα
MW: 40 kDa
1/1000
Ig1: sc-28315
Mouse mono
Prep: 5% Milk
ECL Fluix
t expo = 22.1 seg

Fig 3. D

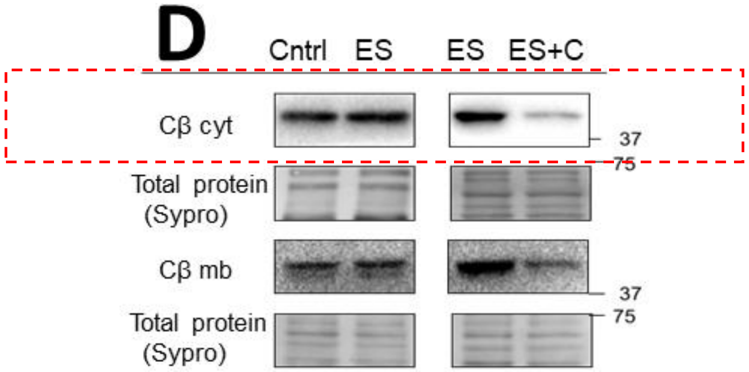

D
Cntrl ES
ES ES+C
Cβ cyt
37
75
Total protein
(Sypro)
Cβ mb
37
75
Total protein
(Sypro)

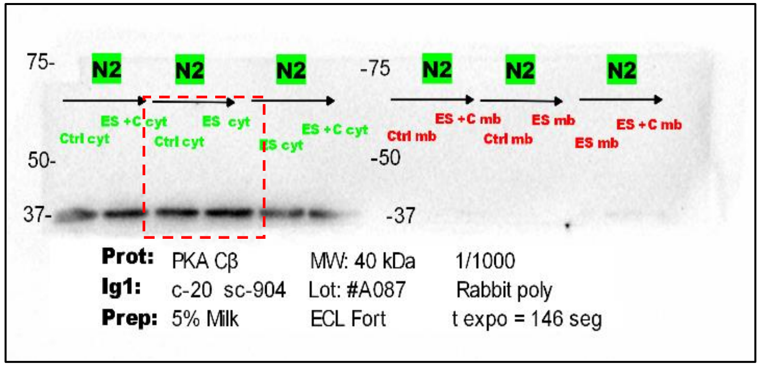

N2
N2
N2
N2
N2
N2
75-
-75
50-
-50
37-
-37
Ctrl cyt
ES +C cyt
Ctrl cyt
ES cyt
ES cyt
ES +C cyt
Ctrl mb
ES +C mb
Ctrl mb
ES mb
ES mb
ES +C mb
Prot: PKA Cβ MW: 40 kDa 1/1000
Ig1: c-20 sc-904 Lot: #A087 Rabbit poly
Prep: 5% Milk ECL Fort t expo = 146 seg

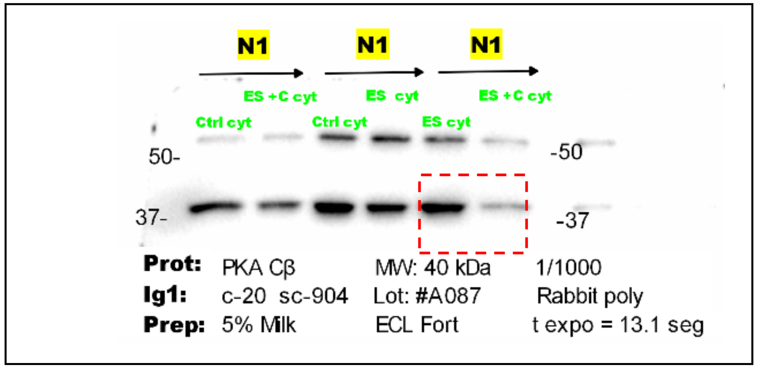

N1
N1
N1
ES +C cyt
ES cyt
ES +C cyt
Ctrl cyt
Ctrl cyt
ES cyt
50-
-50
37-
-37
Prot: PKA Cβ MW: 40 kDa 1/1000
Ig1: c-20 sc-904 Lot: #A087 Rabbit poly
Prep: 5% Milk ECL Fort t expo = 13.1 seg

Fig 3. D

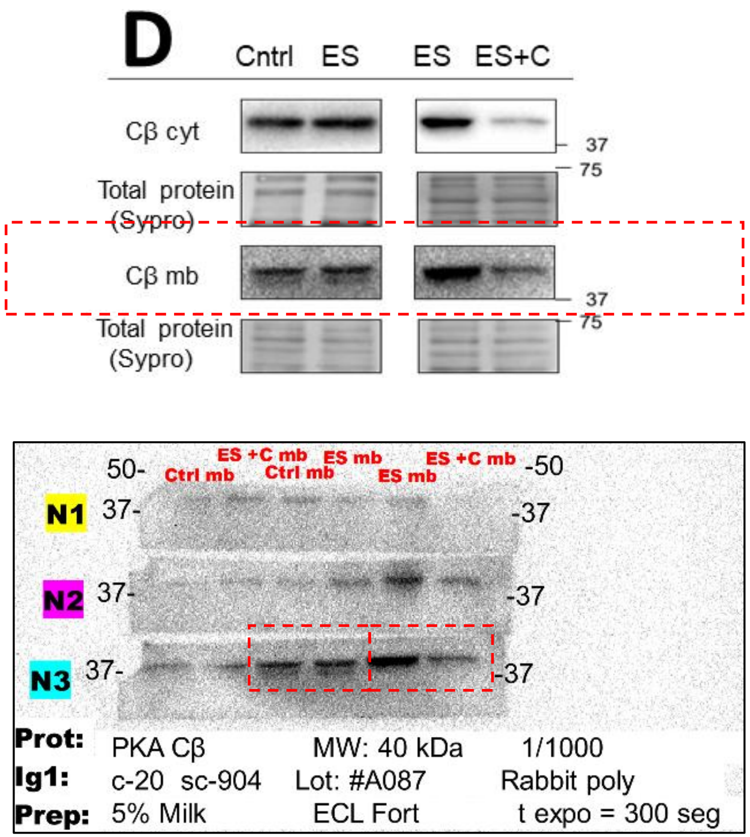
D
Cntrl ES ES ES+C
Cβ cyt
37
75
Total protein (Sypro)
Cβ mb
37
75
Total protein (Sypro)
50-
Ctrl mb
ES +C mb
Ctrl mb
ES mb
ES mb
ES +C mb
-50
N1 37-
-37
N2 37-
-37
N3 37-
-37
Prot: PKA Cβ MW: 40 kDa 1/1000
Ig1: c-20 sc-904 Lot: #A087 Rabbit poly
Prep: 5% Milk ECL Fort t expo = 300 seg

Fig 3. E

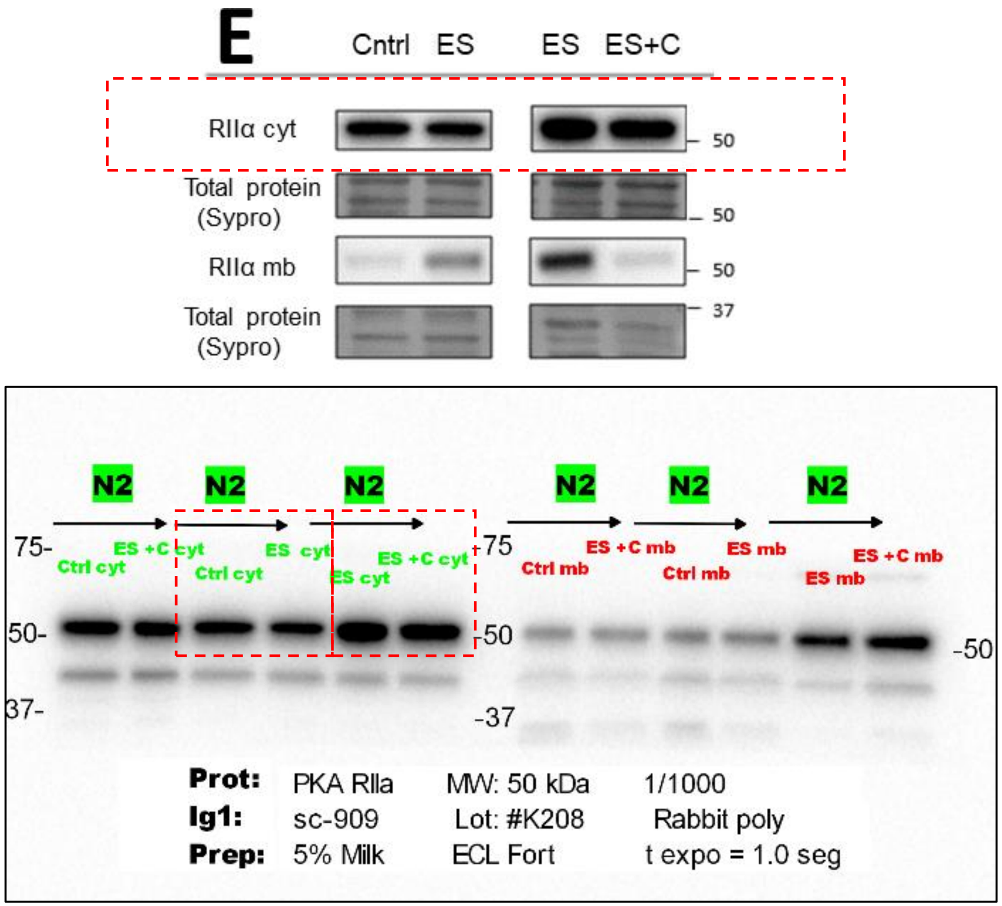
E
Cntrl ES ES ES+C
RIIα cyt
Total protein (Sypro)
RIIα mb
Total protein (Sypro)
50
50
50
37
N2 N2 N2 N2 N2 N2
Ctrl cyt
ES +C cyt
Ctrl cyt
ES cyt
ES cyt
ES +C cyt
Ctrl mb
ES +C mb
Ctrl mb
ES mb
ES mb
ES +C mb
75-
50-
37-
-75
-50
-37
-50
Prot: PKA RIIa MW: 50 kDa 1/1000
Ig1: sc-909 Lot: #K208 Rabbit poly
Prep: 5% Milk ECL Fort t expo = 1.0 seg

Fig 3. E

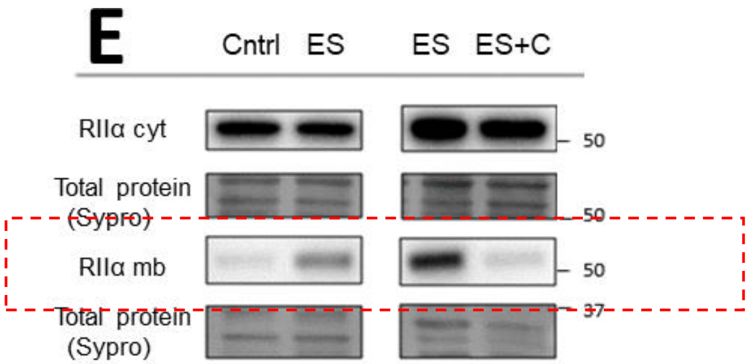
E
Cntrl ES
ES ES+C
RIIα cyt
50
Total protein
(Sypro)
50
RIIα mb
50
Total protein
(Sypro)
37

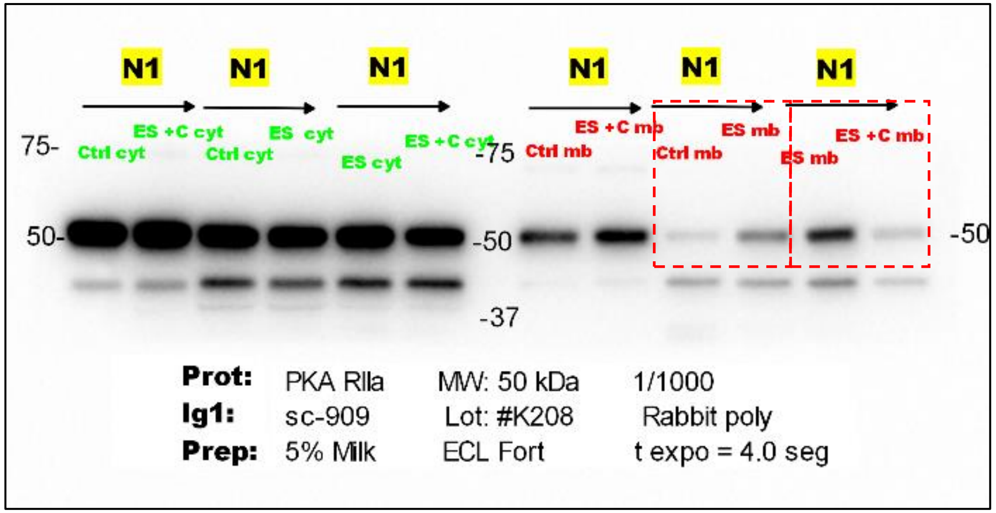
N1
N1
N1
N1
N1
N1
75-
Ctrl cyt
ES +C cyt
Ctrl cyt
ES cyt
ES cyt
ES +C cyt
-75
Ctrl mb
ES +C mb
Ctrl mb
ES mb
ES mb
ES +C mb
50-
-50
-50
-37
Prot: PKA RIIa MW: 50 kDa 1/1000
Ig1: sc-909 Lot: #K208 Rabbit poly
Prep: 5% Milk ECL Fort t expo = 4.0 seg

Fig 3. F

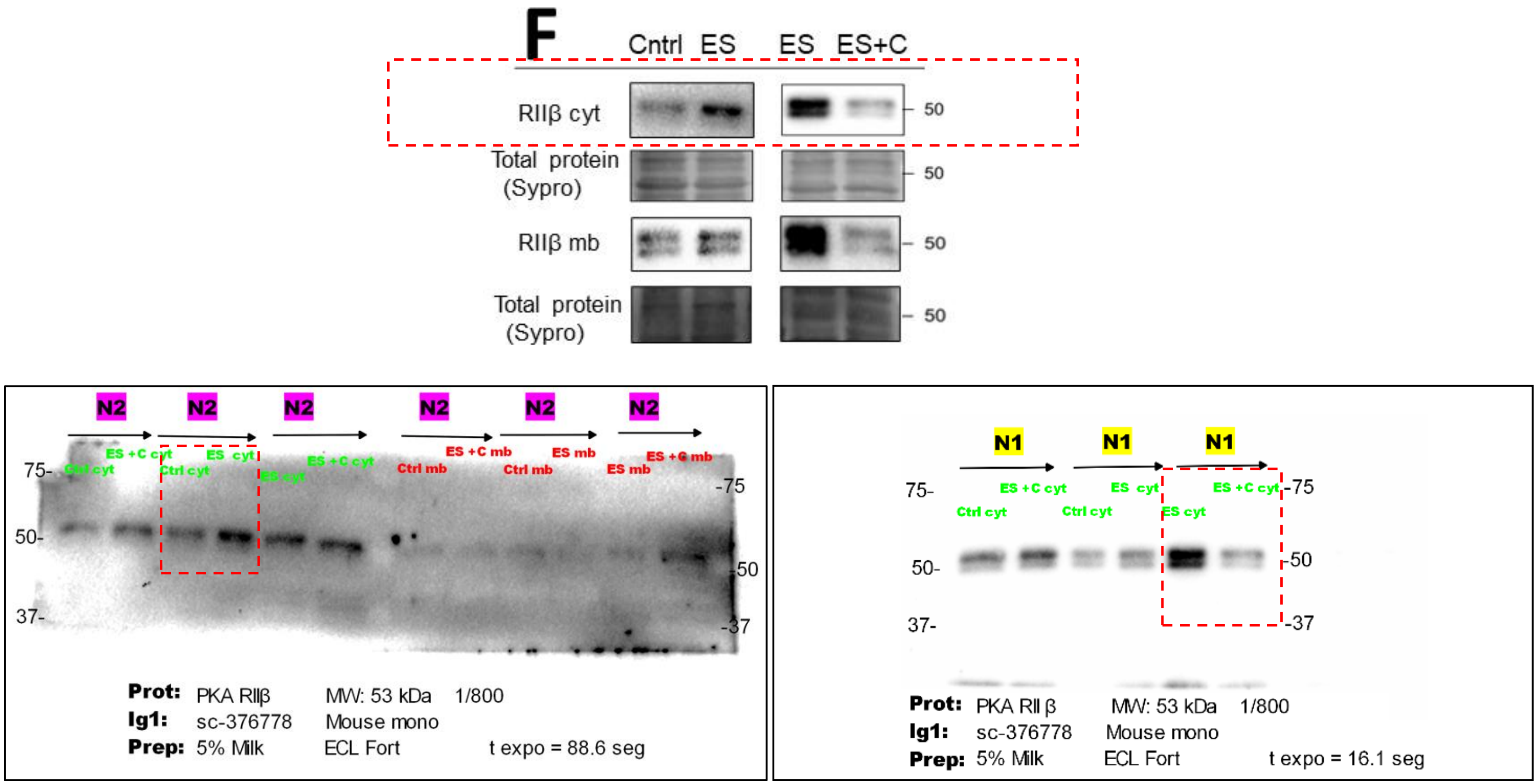
F
Cntrl ES
ES ES+C
RIIβ cyt
Total protein (Sypro)
RIIβ mb
Total protein (Sypro)
50
N2
Ctrl cyt
ES +C cyt
ES cyt
Ctrl mb
ES +C mb
ES mb
75-
50-
37-
Prot: PKA RIIβ MW: 53 kDa 1/800
Ig1: sc-376778 Mouse mono
Prep: 5% Milk ECL Fort t expo = 88.6 seg
N1
Prot: PKA RII β MW: 53 kDa 1/800
Ig1: sc-376778 Mouse mono
Prep: 5% Milk ECL Fort t expo = 16.1 seg

Fig 3. F

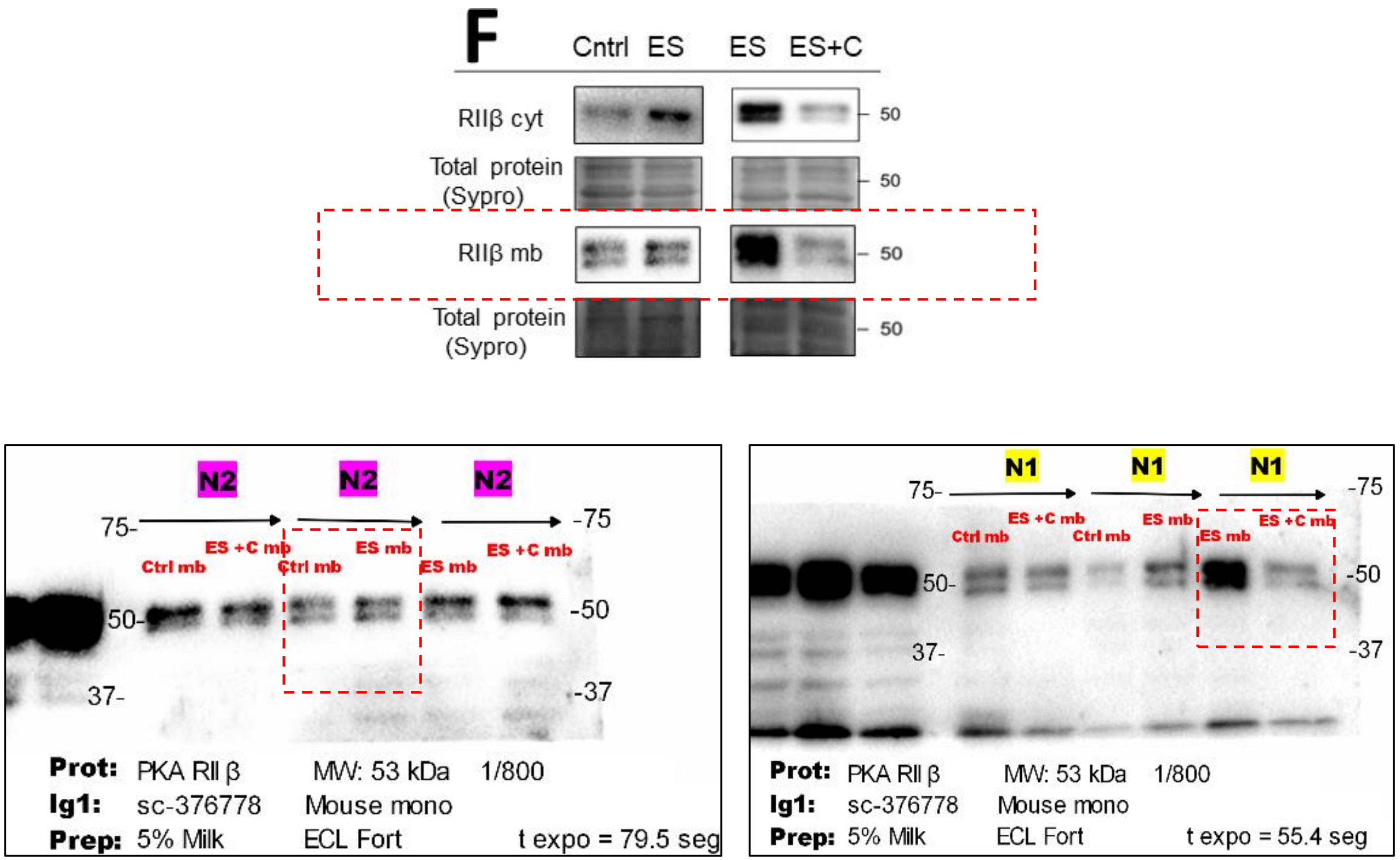
F
Cntrl ES ES ES+C
RIIβ cyt
Total protein (Sypro)
RIIβ mb
Total protein (Sypro)
50
N2
N1
Ctrl mb
ES +C mb
ES mb
75-
50-
37-
Prot: PKA RII β MW: 53 kDa 1/800
Ig1: sc-376778 Mouse mono
Prep: 5% Milk ECL Fort t expo = 79.5 seg
Prot: PKA RII β MW: 53 kDa 1/800
Ig1: sc-376778 Mouse mono
Prep: 5% Milk ECL Fort t expo = 55.4 seg

Fig 3. G

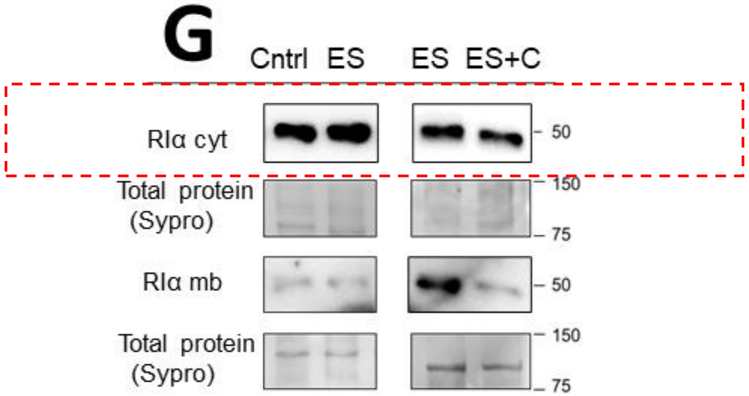
G
Cntrl ES ES ES+C
RIα cyt
50
Total protein (Sypro)
150
75
RIα mb
50
Total protein (Sypro)
150
75

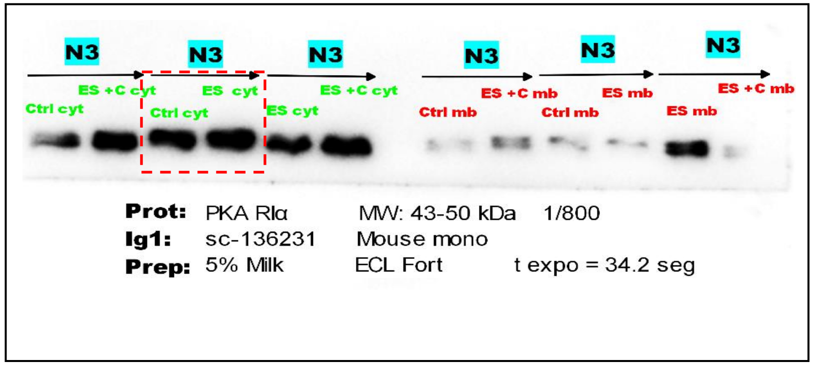
N3 N3 N3 N3 N3 N3
Ctrl cyt
ES +C cyt
Ctrl cyt
ES cyt
ES cyt
ES +C cyt
Ctrl mb
ES +C mb
Ctrl mb
ES mb
ES mb
ES +C mb
Prot: PKA RIα MW: 43-50 kDa 1/800
Ig1: sc-136231 Mouse mono
Prep: 5% Milk ECL Fort t expo = 34.2 seg

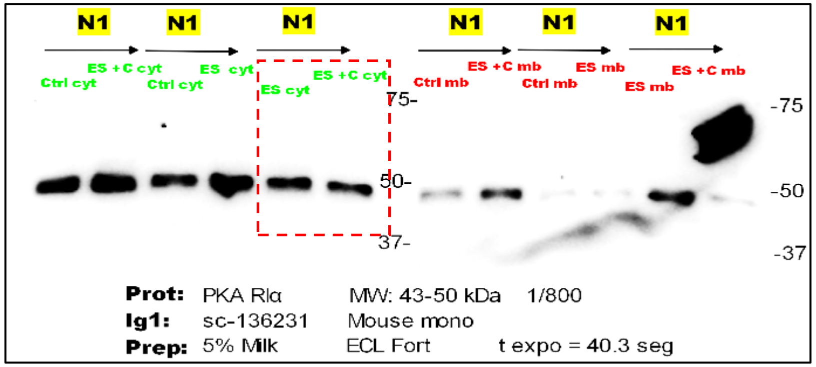
N1 N1 N1 N1 N1 N1
Ctrl cyt
ES +C cyt
Ctrl cyt
ES cyt
ES cyt
ES +C cyt
Ctrl mb
ES +C mb
Ctrl mb
ES mb
ES mb
ES +C mb
75-
50-
37-
-75
-50
-37
Prot: PKA RIα MW: 43-50 kDa 1/800
Ig1: sc-136231 Mouse mono
Prep: 5% Milk ECL Fort t expo = 40.3 seg

Fig 3. G

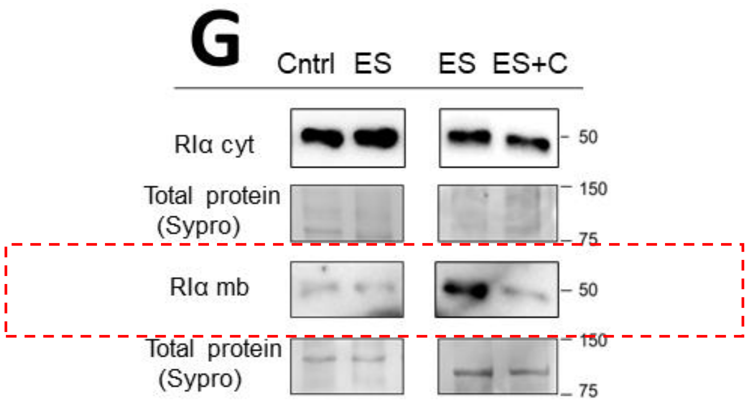
G
Cntrl ES
ES ES+C
RIα cyt
50
Total protein (Sypro)
150
75
RIα mb
50
Total protein (Sypro)
150
75

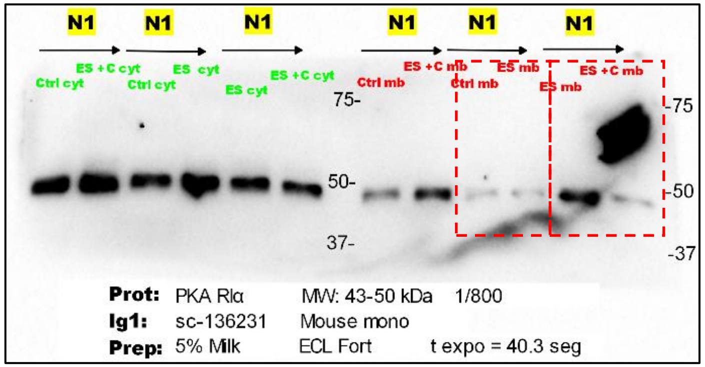
N1
N1
N1
N1
N1
N1
Ctrl cyt
ES +C cyt
Ctrl cyt
ES cyt
ES cyt
ES +C cyt
Ctrl mb
ES +C mb
Ctrl mb
ES mb
ES mb
ES +C mb
75-
50-
37-
-75
-50
-37
Prot: PKA RIα MW: 43-50 kDa 1/800
Ig1: sc-136231 Mouse mono
Prep: 5% Milk ECL Fort t expo = 40.3 seg

Fig 3. H

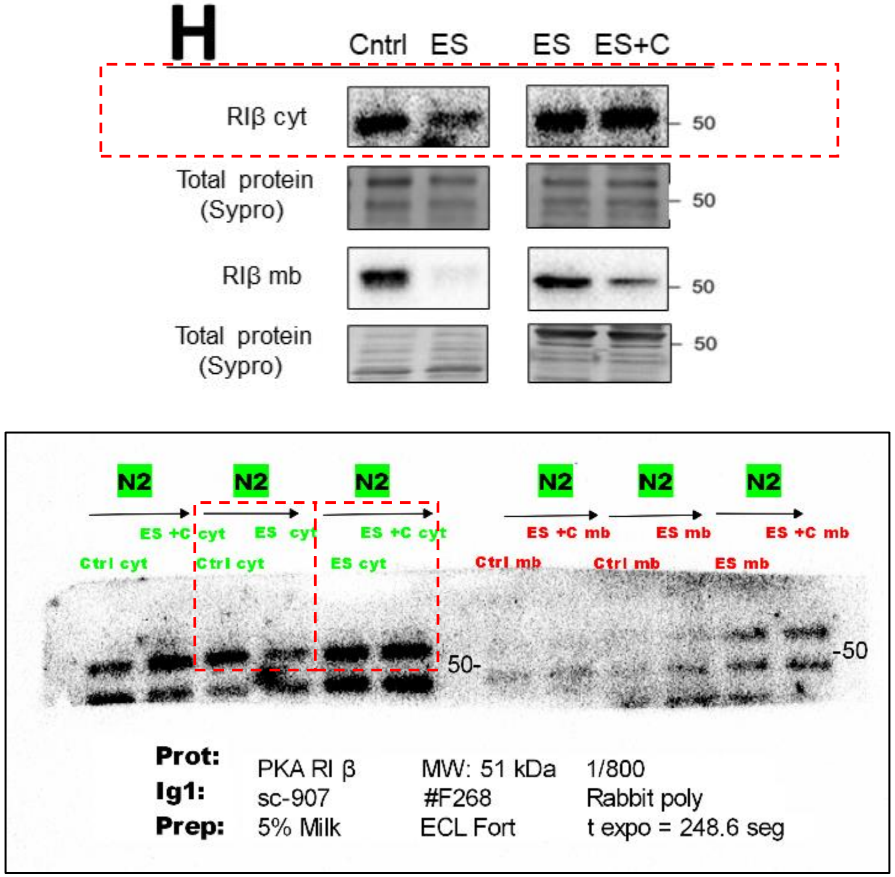
H
Cntrl ES
ES ES+C
RIβ cyt
50
Total protein (Sypro)
50
RIβ mb
50
Total protein (Sypro)
50
N2
ES +C cyt
ES cyt
ES +C cyt
Ctrl cyt
Ctrl cyt
ES cyt
ES +C mb
ES mb
ES +C mb
Ctrl mb
Ctrl mb
ES mb
50-
-50
Prot: PKA RI β MW: 51 kDa 1/800
Ig1: sc-907 #F268 Rabbit poly
Prep: 5% Milk ECL Fort t expo = 248.6 seg

Fig 3. H

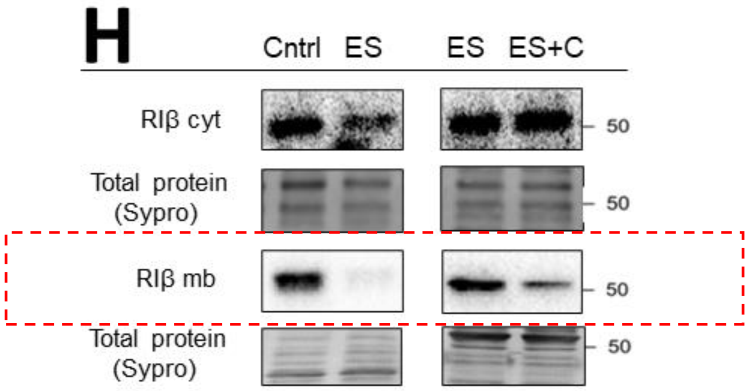

H
Cntrl ES
ES ES+C
RIβ cyt
Total protein (Sypro)
RIβ mb
Total protein (Sypro)
50
50
50
50

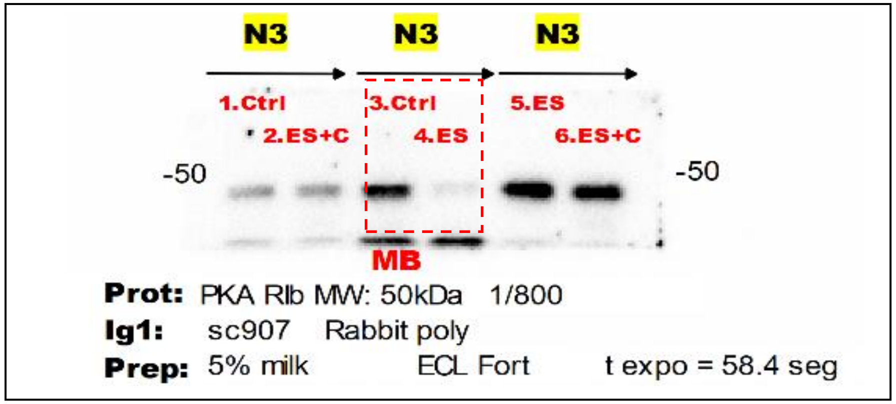

N3
N3
N3
1.Ctrl
2.ES+C
3.Ctrl
4.ES
5.ES
6.ES+C
-50
-50
MB
Prot: PKA RIb MW: 50kDa 1/800
Ig1: sc907 Rabbit poly
Prep: 5% milk ECL Fort t expo = 58.4 seg

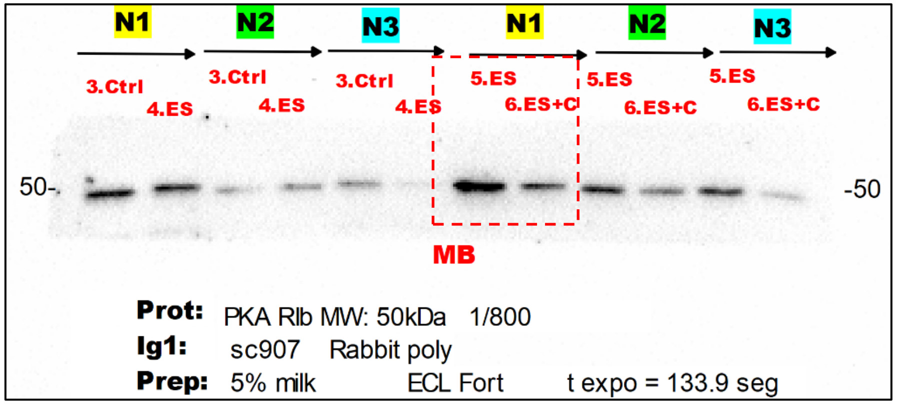

N1
N2
N3
N1
N2
N3
3.Ctrl
4.ES
3.Ctrl
4.ES
3.Ctrl
4.ES
5.ES
6.ES+C
5.ES
6.ES+C
5.ES
6.ES+C
50-
-50
MB
Prot: PKA RIb MW: 50kDa 1/800
Ig1: sc907 Rabbit poly
Prep: 5% milk ECL Fort t expo = 133.9 seg

FIGURE 4

Fig 4. B

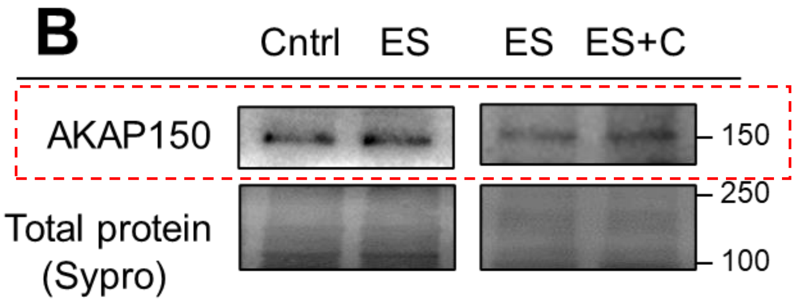

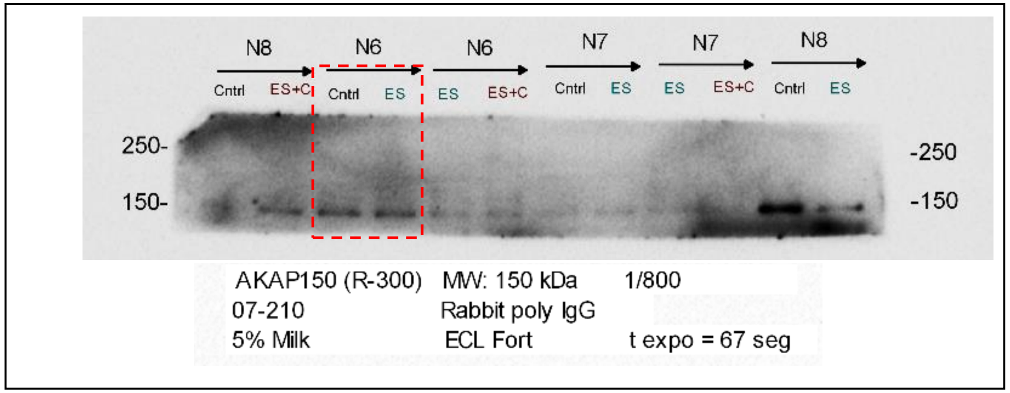

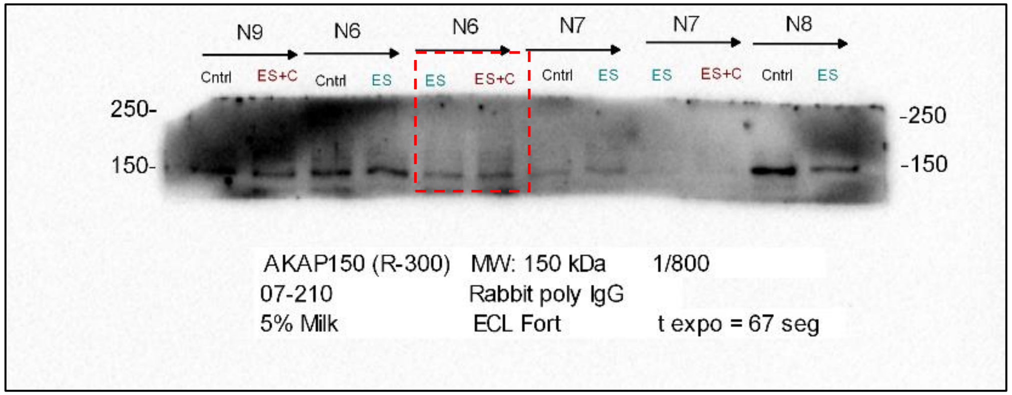

FIGURE 6

Fig 6. A SNAP-25

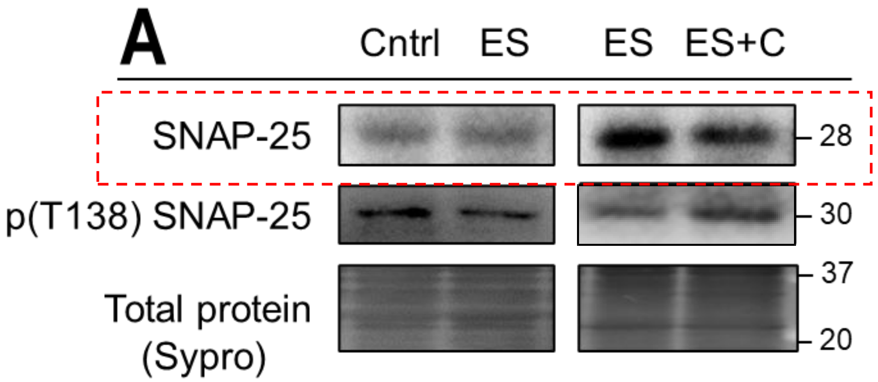

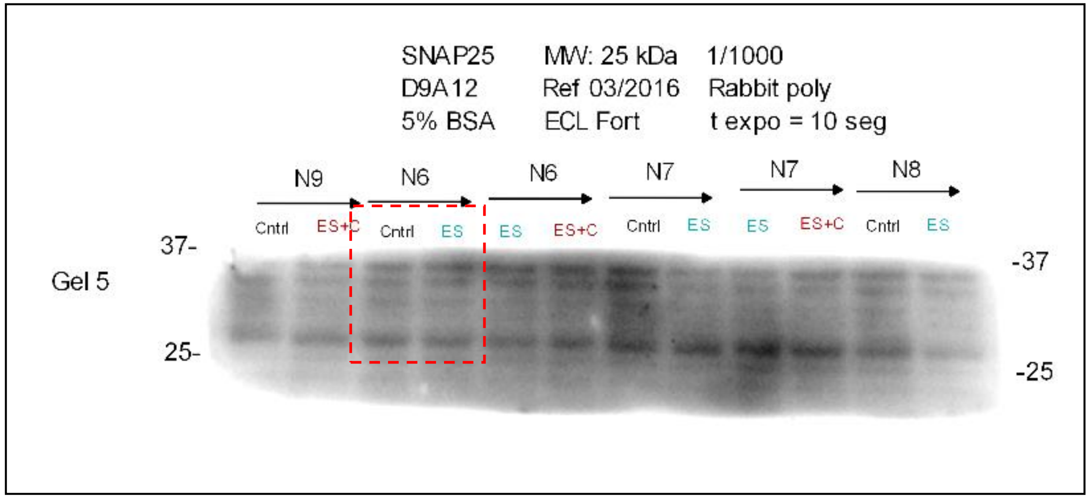

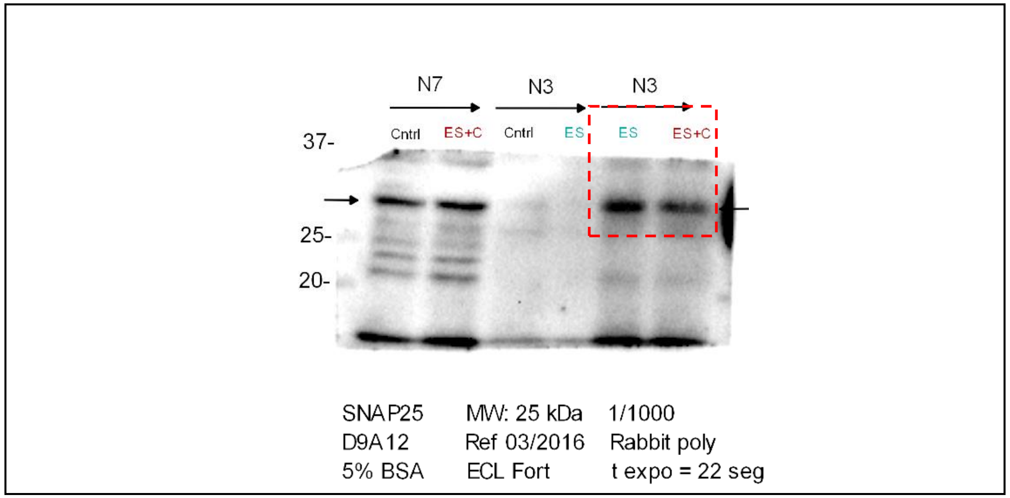

Fig 6. A pSNAP-25

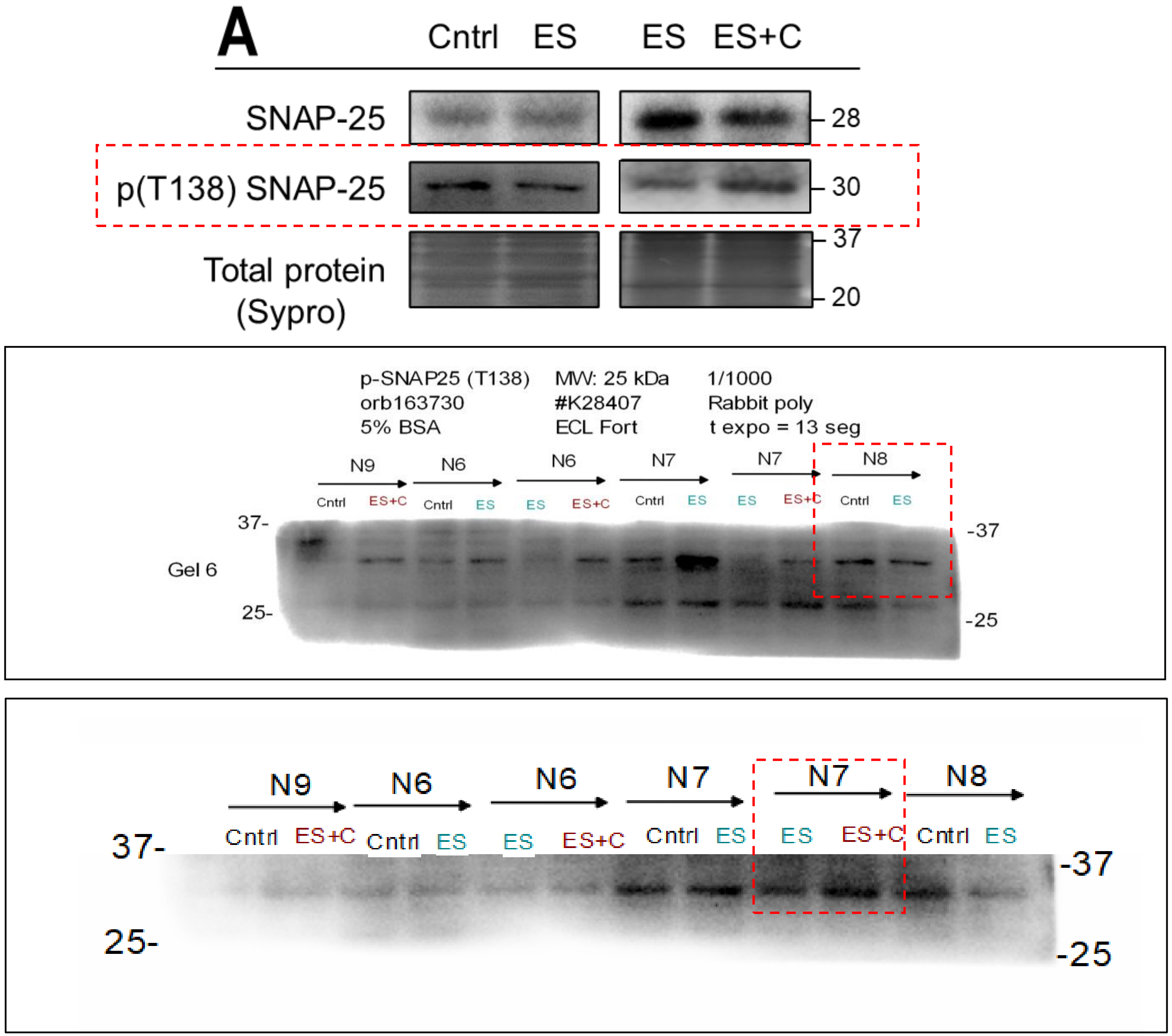

Fig 6. B Synapsin-1

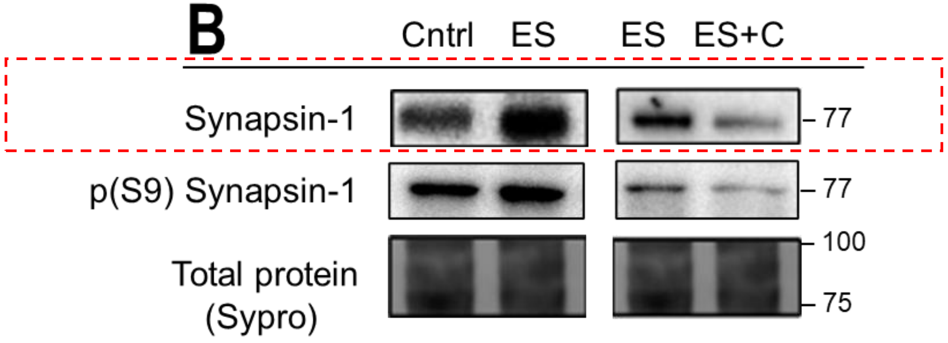

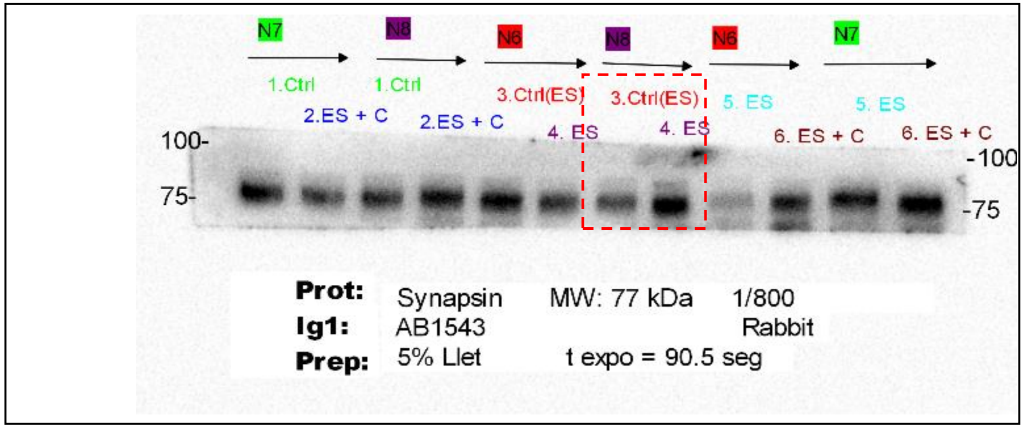

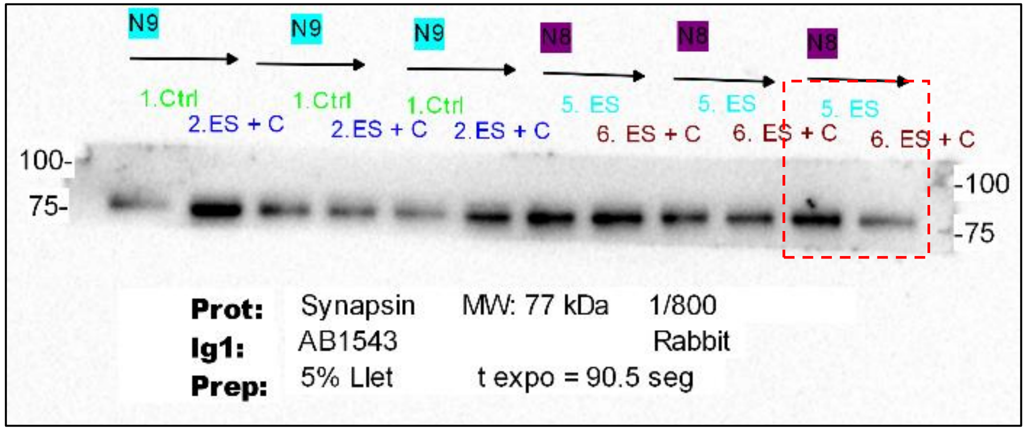

Fig 6. B pSynapsin-1

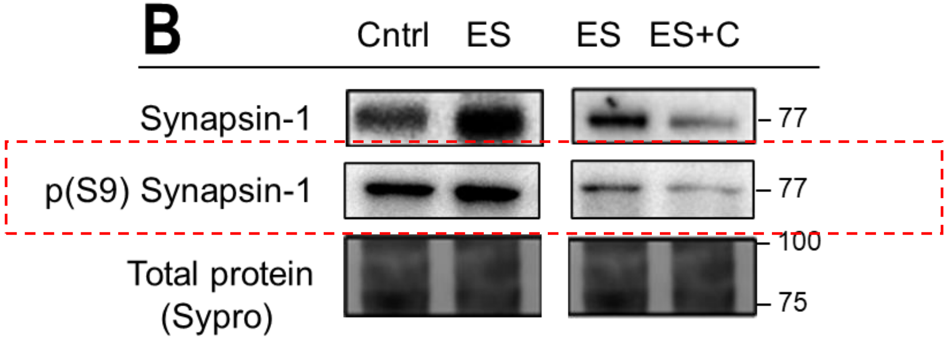

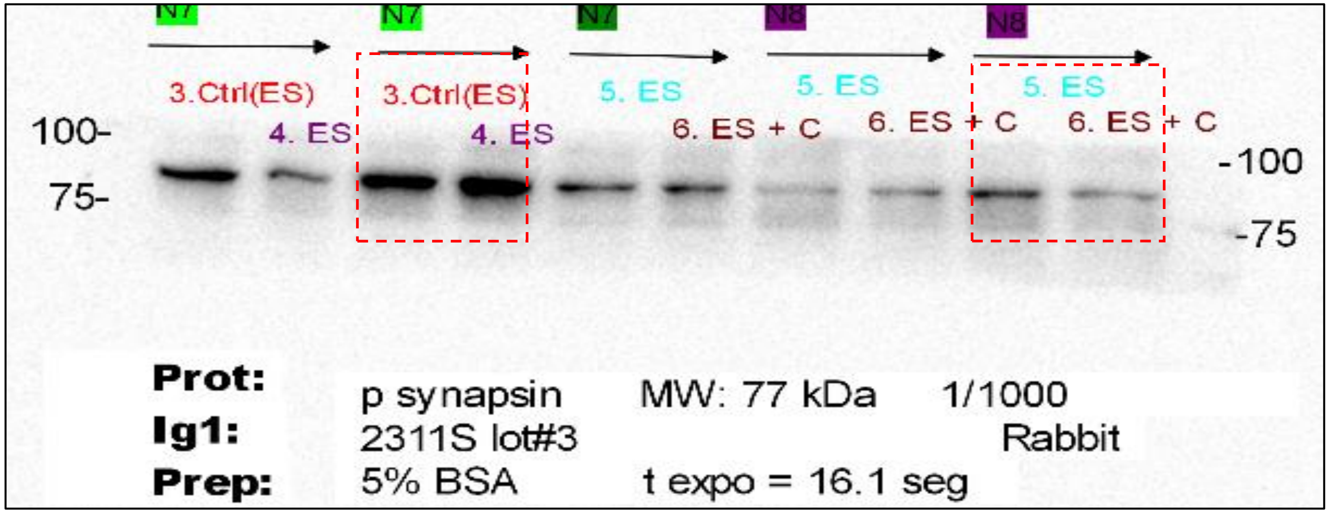

Fig 6. C SNAP-25

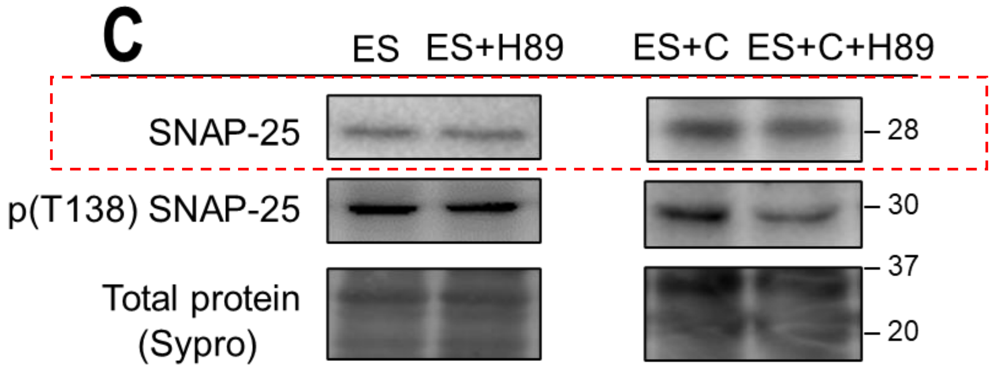

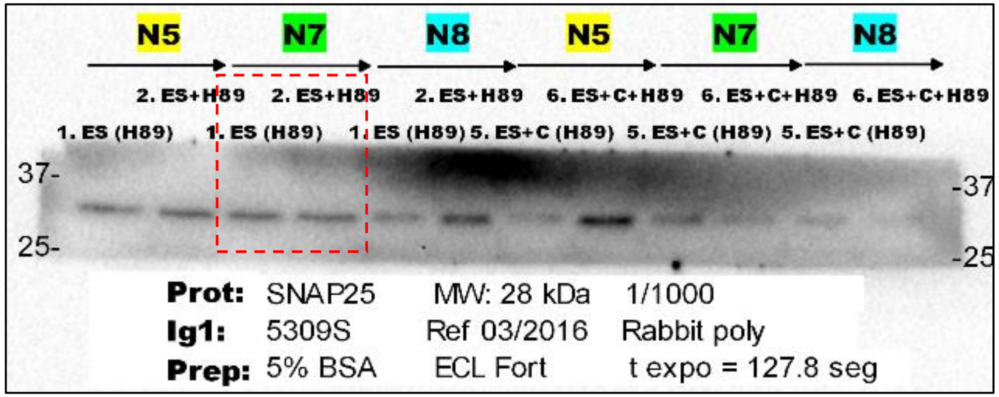

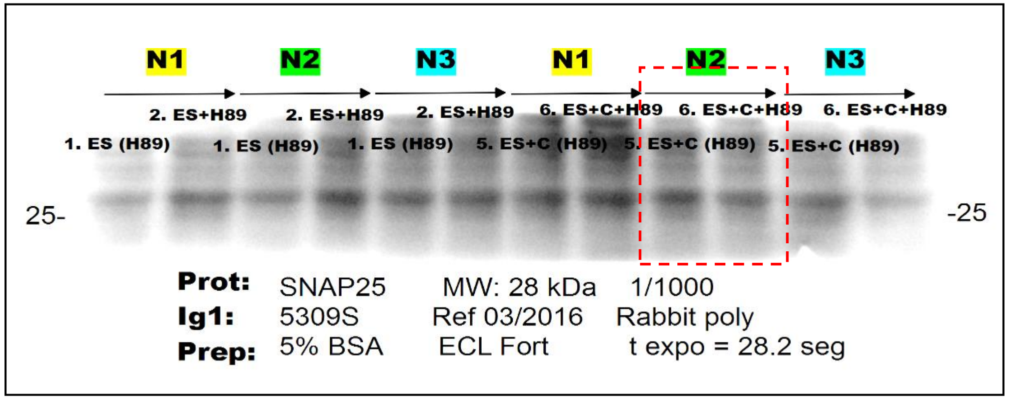

Fig 6. C pSNAP-25

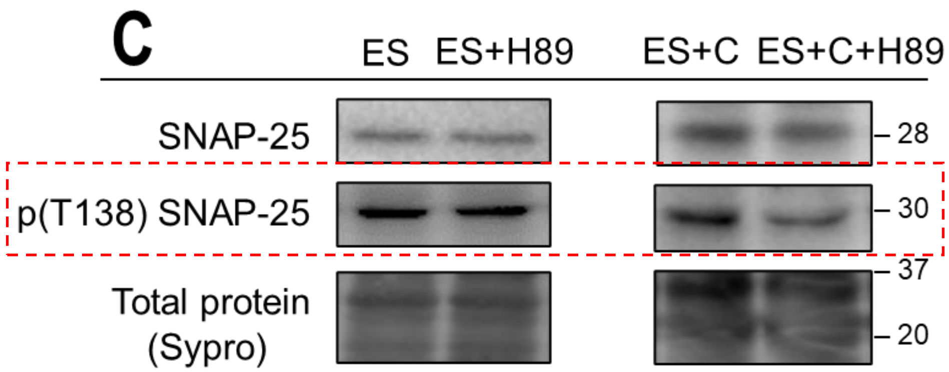

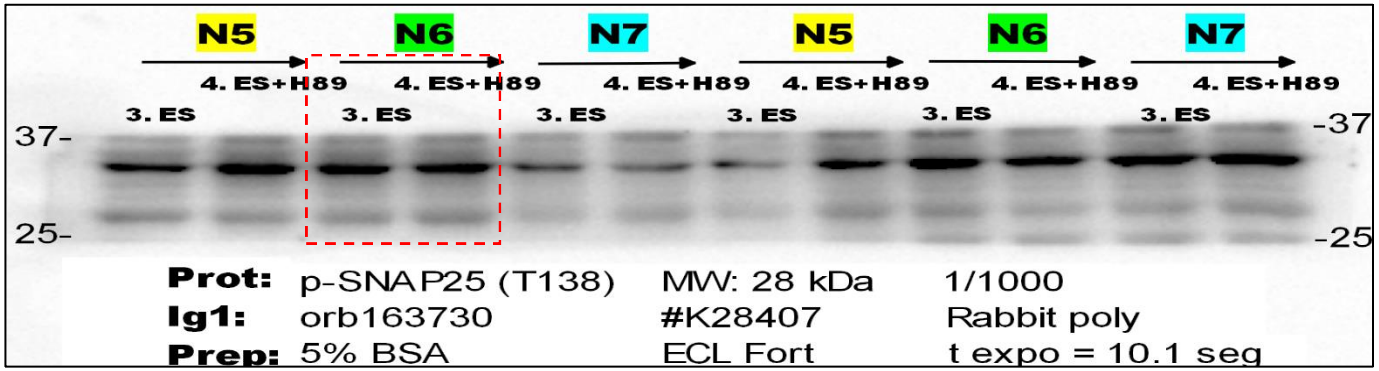

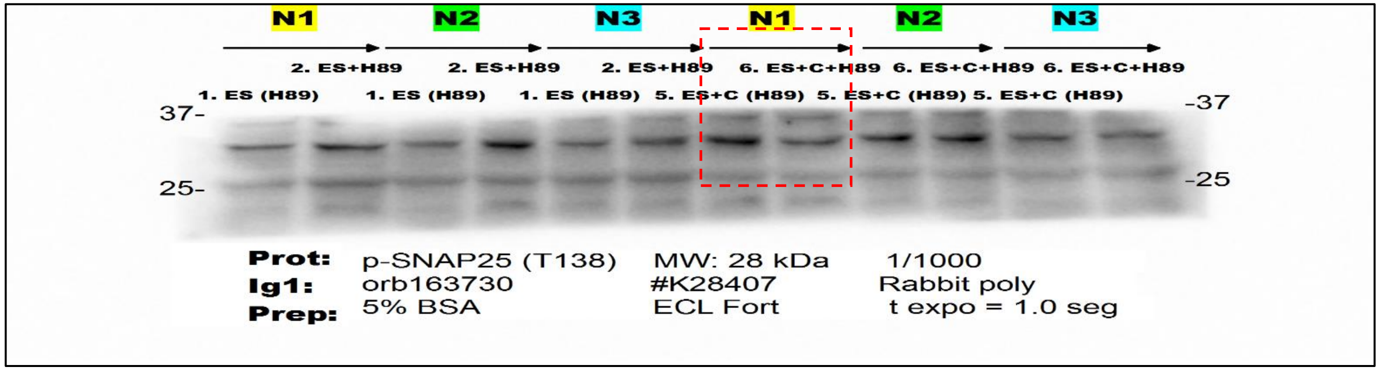

Fig 6. D Synapsin-1

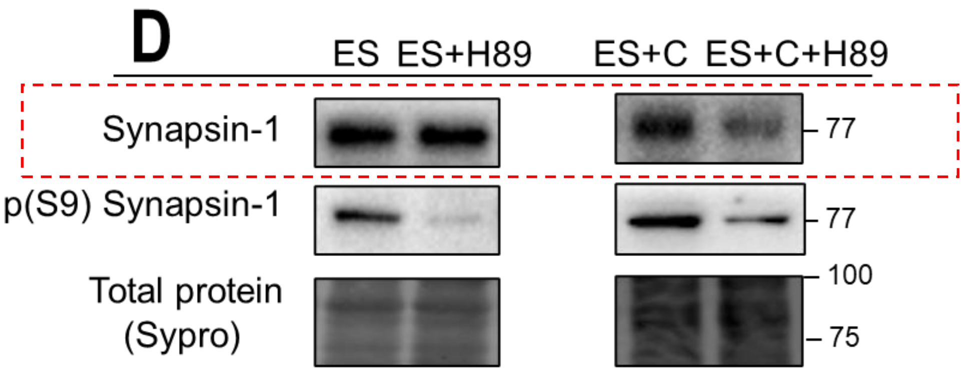

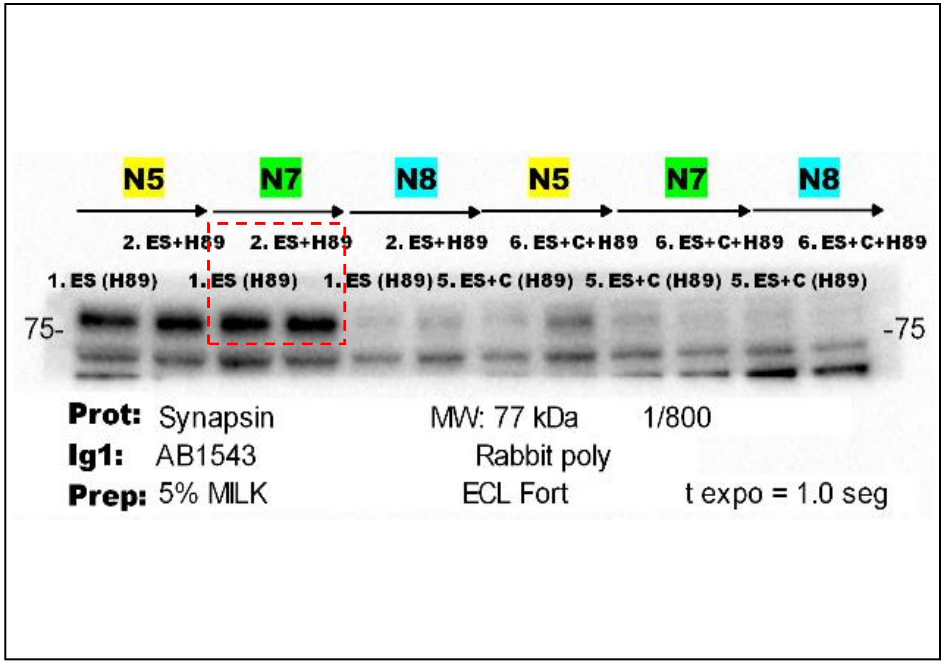

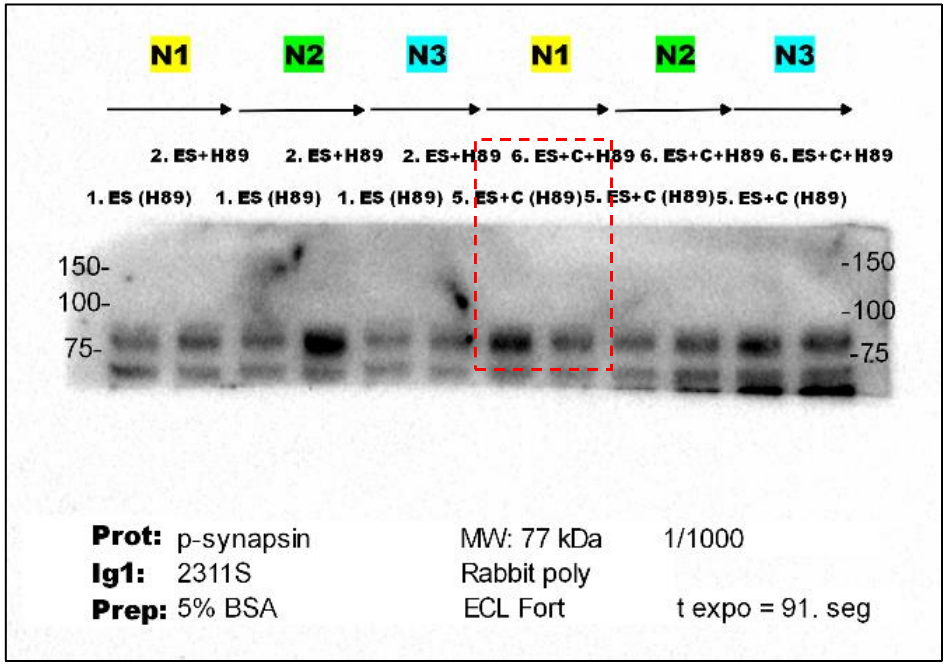

Fig 6. D pSynapsin-1

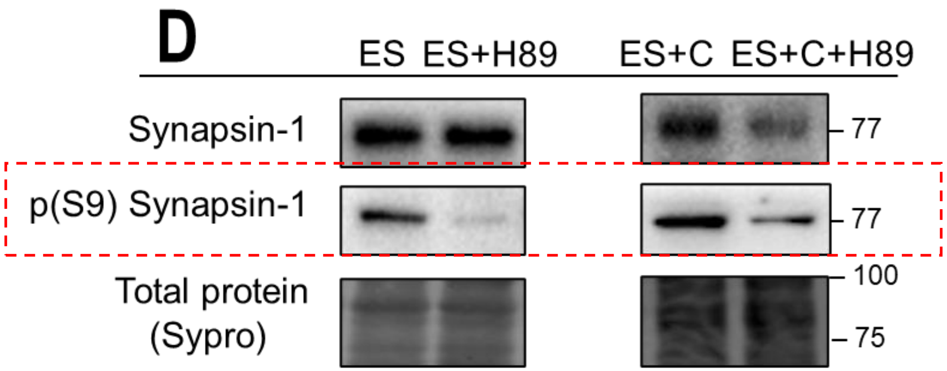

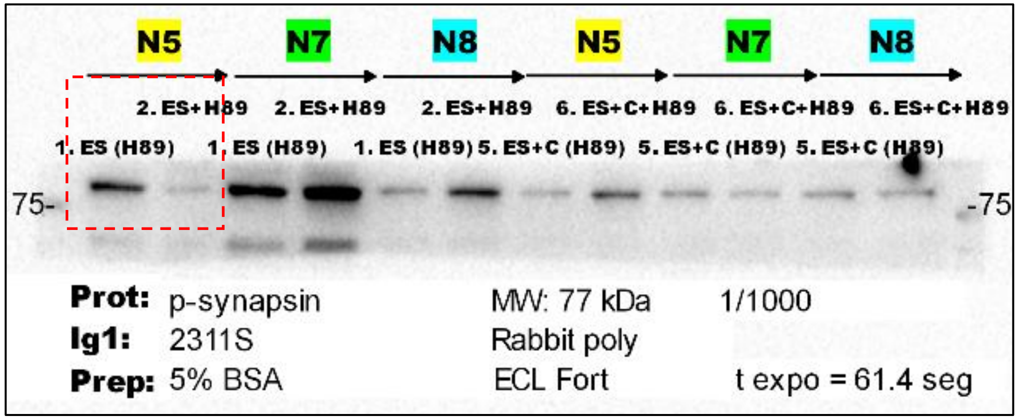

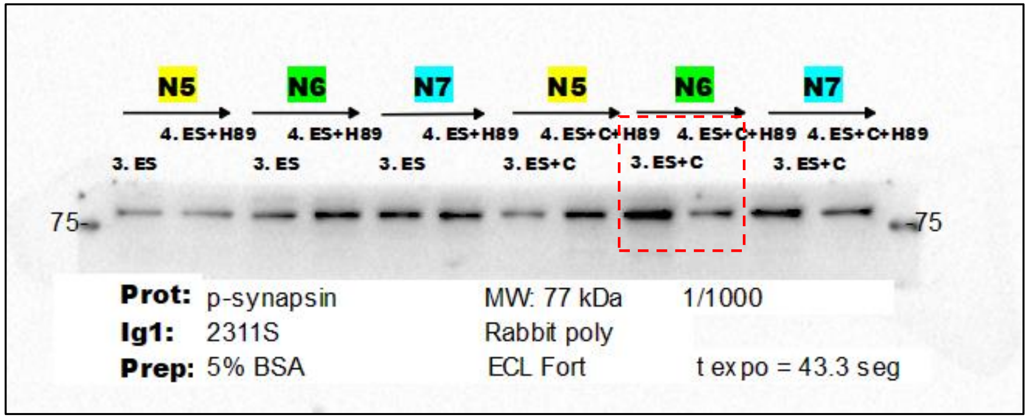

Supplement: Supplementary file 1 — Additional file 1. Original blots. The western blotting pictures were cropped from the original pictures with different conditions but not modified. Each dot in the bars of the graphs is representing the mean result of one animal. [file 11658_2023_431_MOESM1_ESM.pdf]
